# Interventions for intimate partner violence during the perinatal period: A scoping review: A systematic review

**DOI:** 10.1002/cl2.1423

**Published:** 2024-07-15

**Authors:** Olivia Mercier, Sarah Yu Fu, Rachel Filler, Alexie Leclerc, Kari Sampsel, Karine Fournier, Mark Walker, Shi Wu Wen, Katherine Muldoon

**Affiliations:** ^1^ Faculty of Medicine University of Ottawa Ottawa Ontario Canada; ^2^ Clinical Epidemiology Program Ottawa Hospital Research Institute Ottawa Ontario Canada; ^3^ Department of Emergency Medicine The Ottawa Hospital Ottawa Ontario Canada; ^4^ Health Sciences Library University of Ottawa Ottawa Ontario Canada; ^5^ Department of Global Health and Internationalization University of Ottawa Ottawa Ontario Canada; ^6^ Department of Obstetrics and Gynecology University of Ottawa Ottawa Ontario Canada; ^7^ Department of Obstetrics, Gynecology and Newborn Care The Ottawa Hospital Ottawa Ontario Canada; ^8^ School of Epidemiology and Public Health University of Ottawa Ottawa Ontario Canada; ^9^ Children's Hospital of Eastern Ontario Ottawa Ontario Canada

**Keywords:** gender‐based violence, interventions, intimate partner violence, perinatal, pregnancy, scoping review, violence

## Abstract

**Background:**

Intimate partner violence (IPV) is a prevalent global health problem. IPV that occurs before pregnancy often continues during the perinatal period, resulting in ongoing violence and many adverse maternal, obstetrical, and neonatal outcomes.

**Objectives:**

This scoping review is designed to broadly capture all potential interventions for perinatal IPV and describe their core components and measured outcomes.

**Search Methods:**

We conducted a search for empirical studies describing IPV interventions in the perinatal population in June 2022. The search was conducted in MEDLINE, EMBASE, PsycInfo, CINAHL, Cochrane Central Register of Controlled Trials, Web of Science, Applied Social Sciences Index & Abstracts, ClinicalTrials.gov and MedRxiv. Hand searching of references from select articles was also performed.

**Selection Criteria:**

Included studies described an intervention for those experiencing IPV during the perinatal period, including 12 months before pregnancy, while pregnant or in the 12 months post‐partum. The search encompassed January 2000 to June 2022 and only peer‐reviewed studies written in either English or French were included. Included interventions focused on the survivor exposed to IPV, rather than healthcare professionals administering the intervention. Interventions designed to reduce IPV revictimization or any adverse maternal, obstetrical, or neonatal health outcomes as well as social outcomes related to IPV victimization were included.

**Data Collections and Analysis:**

We used standard methodological procedures expected by The Campbell Collaboration.

**Main Results:**

In total, 10,079 titles and abstracts were screened and 226 proceeded to full text screening. A total of 67 studies included perinatal IPV interventions and were included in the final sample. These studies included a total of 27,327 participants. Included studies originated from 19 countries, and the majority were randomized controlled trials (*n* = 43). Most studies were of moderate or low quality. Interventions included home visitation, educational modules, counseling, and cash transfer programs and occurred primarily in community obstetrician and gynecologist clinics, hospitals, or in participants' homes. Most interventions focused on reducing revictimization of IPV (*n* = 38), improving survivor knowledge or acceptance of violence, knowledge of community resources, and actions to reduce violence (*n* = 28), and improving maternal mental health outcomes (*n* = 26). Few studies evaluated the effect of perinatal IPV interventions on obstetrical, neonatal or child health outcomes.

**Authors' Conclusion(s):**

The majority of intervention studies for perinatal IPV focus on reducing revictimization and improving mental health outcomes, very few included obstetrical, neonatal, and other physical health outcomes. Future interventions should place a larger emphasis on targeting maternal and neonatal outcomes to have the largest possible impact on the lives and families of IPV survivors and their infants.

## 1 PLAIN LANGUAGE SUMMARY

Existing interventions for intimate partner violence (IPV) during the perinatal period focus on revictimization and maternal mental health as outcomes; future studies should assess obstetrical and neonatal outcomes.

### 1.1 The review in brief

Existing interventions designed to target IPV during the perinatal period are diverse. Most commonly, interventions consisted of an educational component or counseling provided by healthcare workers. Interventions often focused on reducing revictimization and improving maternal mental health (e.g., depression, post‐traumatic stress disorder [PTSD], etc.). Few studies evaluated obstetrical (e.g., gestational diabetes, miscarriage, stillbirth, etc.), neonatal (e.g., neonatal intensive care unit admission, etc.) and child (e.g., growth pattern) health outcomes.

### 1.2 What is this review about?

Approximately 1 in 3 women globally experience some form of IPV during their lifetimes. Studies have shown that for those who experience IPV before pregnancy, IPV continues during the perinatal period. The perinatal period starts 12 months preconception, continues through pregnancy, and extends until 12 months post‐partum. IPV during the perinatal period results in many adverse outcomes for both the mother (or person experiencing the pregnancy) and the infant. It is critical to identify those exposed to IPV and intervene during the perinatal period.

### 1.3 What is the aim of this review?

This Campbell scoping review describes the existing interventions for IPV during the perinatal period, including their core components and measured outcomes. The review summarizes evidence from 67 studies.

### 1.4 What are the main findings of this review?

#### 1.4.1 What studies are included?

Included studies evaluated interventions for IPV during the perinatal period. Studies were included if they were peer‐reviewed, written in English or French, and evaluated an intervention for those who were currently experiencing perinatal IPV. Study inclusion was not limited by study design. A total of 67 studies were included.

#### 1.4.2 How has this intervention worked?

A wide variety of perinatal IPV interventions have been conducted around the world. The most common study designs are randomized controlled trials (RCTs), in addition to pre‐test post‐test, cohorts, case controls, mixed methods, and qualitative designs. Intervention types include cash transfer programs, home visitation programs, educational modules, and counseling. Most interventions feature a component of education or counseling, and many interventions include multiple components.

Included studies evaluated a wide range of outcomes including: revictimization of IPV, maternal mental health, maternal physical health, obstetrical health, neonatal and child health, family‐related outcomes, and self‐esteem related outcomes (i.e., acceptance of IPV, safety behaviors, and learned helplessness). Many studies also measured patient acceptability of the intervention and feasibility. Overall, IPV revictimization and maternal mental health were measured most frequently. Few studies measured obstetrical, neonatal, and child outcomes.

### 1.5 What do the findings of this review mean?

There are many different interventions for perinatal IPV which aim to reduce violence and improve outcomes associated with maternal health. Perinatal IPV interventions can occur in many settings, including clinical and community‐based settings. There are different types of interventions including education sessions, counseling, and home visitation programs.

We did not comment on the effectiveness of each intervention, and this is important data to gather in future research. Future research should include metrics on obstetrical and neonatal health outcomes to help document and understand how violence affects pregnancy and child development, and provide the greatest possible impact on the lives of survivors and their families.

### 1.6 How up‐to‐date is this review?

The review authors searched for studies published up to June of 2022. This Campbell Scoping Review was published in 2024.

## 2 BACKGROUND

### 2.1 The problem, condition, or issue

IPV is a prevalent global health problem that affects 1 in 3 women, 1 in 10 men, and 1 in 2 trans or non‐binary people (World Health Organization, 2023a; Centers for Disease Control and Prevention, 2023; Peitzmeier et al., 2020). While violence affects people of all ages and genders, IPV comes from known perpetrators such as intimate partners, family, friends, employers, or acquaintances (World Health Organization, 2023b). IPV includes any form of physical abuse, sexual abuse, psychological abuse, or controlling behaviors by a current or former intimate partner (World Health Organization, 2023b). IPV is associated with adverse maternal mental health outcomes (e.g., depression, PTSD, anxiety, suicidality, self‐harm, etc.) and maternal physical health outcomes (e.g., chronic health conditions, functional health, etc.) (Dillon et al., 2013; Lagdon et al., 2014).

Many studies have shown that IPV can increase during the perinatal period, defined as the 12 months before pregnancy, during pregnancy, and 12 months post‐partum (Jasinski, 2004; Van Parys et al., 2014). Potential reasons for the increase during the perinatal period include loss of income during parental leave, sleep deprivation, relationship strain, hormonal changes, and the stress of an unplanned pregnancy (Boyacioglu et al., 2021). However, studies have also shown that pregnancy can be a time of protection against physical IPV, and physical violence can decrease as compared to pre‐pregnancy levels (Brownridge et al., 2011; Saltzman et al., 2003). Similarly, studies have shown that psychological IPV can decrease during pregnancy as compared to pre‐pregnancy levels (Brownridge et al., 2011; Islam et al., 2018). The current literature demonstrates that the strongest risk factor for physical IPV during pregnancy is violence before pregnancy, and the strongest risk factor for physical IPV during the post‐partum period is violence during pregnancy (Martin, 2001). While unwanted pregnancy is a risk factor for IPV, pregnancy alone is not a risk indicator for IPV for women who have not experienced IPV before pregnancy (Goodwin et al., 2000; Martin et al., 2001; Yakubovich et al., 2018).

Estimates of physical IPV alone during pregnancy range from 3.7% to 9% (Hahn et al., 2018). However, prevalence of perinatal IPV is likely much higher when expanded to include psychological and sexual violence, as these forms of violence are consistently underreported and inadequately assessed (Hahn et al., 2018). IPV during the perinatal period can increase obstetrical and neonatal adverse outcomes including fetal growth restriction, placental abruption, low birth weight, preterm birth, and perinatal death (including stillbirth and neonatal death) (Alhusen et al., 2015). In extreme cases, it can lead to maternal homicide as reported by the Canadian Femicide Observatory for Justice and Accountability (Stewart et al., 2017).

Universal screening can increase the identification of IPV, however, without providing adequate assistance and support, it is not recommended and, in some cases, considered unethical (World Health Organization, 2023c). A Cochrane review concluded that while universal screening can increase identification of IPV, universal screening itself does not reduce adverse outcomes associated with IPV and therefore must be followed by an effective intervention (O'Doherty et al., 2015). As an alternative to universal screening, the World Health Organization recommends healthcare professionals inquire about IPV when assessing conditions that may be caused or complicated by IPV, such as traumatic injury with vague or implausible explanations (World Health Organization, 2023c). There is a need for effective interventions to improve obstetrical and neonatal health outcomes for survivors exposed to IPV during the perinatal period. During the perinatal period, individuals are highly engaged with the health system and have frequent encounters for prenatal care, labor and delivery, and post‐partum follow‐up. As such, during the perinatal period, a range of healthcare professionals who provide services and support to this population are ideally positioned to identify IPV and intervene.

### 2.2 Description of the interventions

This scoping review synthesizes the literature on interventions designed to support survivors exposed to IPV during the perinatal period. These interventions have been designed to reduce harms associated with perinatal IPV including (but not limited to): revictimization, physical or mental harm, and adverse obstetrical and neonatal outcomes. These interventions include: (i) home visitation, (ii) cash transfer, (iii) counseling, (iv) education, or a combination thereof. The interventions are delivered individually or in group format, are administered directly to the survivor of IPV, and may take place in either a healthcare or community setting.

### 2.3 How the intervention might work

#### 2.3.1 Home visitation

Home visitation interventions are conducted by a trained provider who visits a survivor at their home to provide support. In the context of survivors of IPV, the goals of home visitation are to identify IPV, increase safety, improve mental and physical health, reduce revictimization, and improve the family dynamics and relationships to create a safer environment during the perinatal period and beyond. Interventions in the form of home visitation are particularly beneficial during the perinatal period, as survivors adjust to the increased demands of parenthood, because they are not required to leave their homes to participate in the intervention.

Often, IPV‐specific home visitation interventions are integrated within an existing home visitation program that has broad health promotion goals to improve overall maternal health in pregnancy, child health growth and development, and maternal/parent life course trajectories. For example, the Domestic Violence Enhanced Home Visitation Program (DOVE) is an empowerment intervention integrated within existing home visitation programs in the United States (Sharps et al., 2016). DOVE is composed of six home visits scheduled during pregnancy and during the post‐partum period. Each session included the home visitor discussing safety planning tailored to the participant, available resources for IPV, a national hotline, and more (Sharps et al., 2016).

Addition of an intervention to target IPV to a pre‐existing program increases adherence and makes the intervention more accessible for participants. However, addressing IPV in home visitation programs can be challenging as the intervention occurs in the home, and the perpetrator is often present (Jack et al., 2023). As such, home visitation programs that target IPV are only possible for survivors who do not live with their perpetrator, or for survivors who can reliably gain privacy from their perpetrator during the home visit.

#### 2.3.2 Cash transfer

Cash transfer programs have been shown to have several benefits for reducing IPV and promoting gender equality. These include economic empowerment, reducing stress and tension related to financial strain, improving gender roles and norms by financially valuing women's work and contributions, improving access to education and health care, and increasing social protections (Bonilla et al., 2017; Fuller et al., 2022). For example, LEAP 1000 is a cash transfer program implemented in Ghana providing biweekly unconditional cash transfers and health insurance to women who are pregnant or have a child under the age of 12 months. The program was designed to address poverty‐related drivers of poor nutritional outcomes within the first 1000 days of a child's life, including reduction of IPV (Peterman et al., 2022).

#### 2.3.3 Counseling

A wide variety of therapeutic approaches have been used to provide support to survivors of perinatal IPV, such as cognitive behavioral therapy (CBT) (Mantler et al., 2022), motivational interviewing (Sapkota et al., 2022), and problem‐solving therapy (Nakku et al., 2021). Counseling sessions are often integrated into pre‐existing prenatal care visits or added as a supplementary program. Intervention length can vary, from a one‐time counseling session to regular sessions over the course of multiple years.

#### 2.3.4 Education

Education sessions for perinatal IPV can be a useful tool for increasing awareness, promoting prevention, providing support, and reducing harms. Education topics can include the identification of warning signs and risk factors for violence, developing strategies to recognize warning signs, safety planning strategies, and local resources available for survivors. Education sessions may be delivered in individual format, group format, or self‐study format and may be delivered in‐person or using a computerized method.

### 2.4 Why it is important to do this review

To date, there is no scoping review on interventions for perinatal IPV. There are five systematic reviews that examine interventions for IPV during the perinatal period. In total, these reviews include 23 unique interventions, and two are restricted exclusively to the prenatal period (Jahanfar et al., 2014; O'Reilly et al., 2010), while three are expanded to include pregnant and post‐partum women (Daley et al., 2020; Reyes et al., 2021; Van Parys et al., 2014). Existing reviews were limited to low‐ and middle‐income countries (Daley et al., 2020) and based on intervention type (Reyes et al., 2021; Van Parys et al., 2014). Additionally, two systematic reviews were limited to RCTs (Jahanfar et al., 2014; Van Parys et al., 2014). None of the existing systematic reviews included detailed information on the interventions themselves.

There are two thematic reviews written on IPV interventions for the general population, however they are limited in respect to intervention types. One review explores only qualitative studies that evaluate nurse home visitation program interventions (Adams et al., 2023) while the other includes only advocacy interventions or interventions that include an advocacy component (Rivas et al., 2019). Therefore, there is no description of intervention outcomes across various intervention types. Additionally, when reviews are limited to RCTs only, innovative intervention designs that lack resources to employ a RCT are automatically excluded.

Overall, while there are existing reviews on perinatal IPV interventions, they are limited. There is a need for a review to cover the entire perinatal period (defined here as 12 months pre‐conception to 12 months post‐partum), and include a range of study designs (e.g., quantitative, qualitative) and intervention types (e.g., psychotherapy, safety planning, cash transfer programs) across all disciplines (e.g., medicine, psychology, sociology). Additionally, providing an overview of all existing interventions for IPV during the perinatal period and identifying the wide range of outcomes that have been identified will help to inform future intervention designs and aims. Ultimately, this will contribute to the development of effective interventions that will reduce IPV victimization during the perinatal period, and its associated adverse outcomes.

## 3 OBJECTIVES

The objectives of this scoping review are to:

1. Identify and describe the range of existing interventions that have been used globally to support survivors exposed to IPV during the perinatal period;
2. Describe the core components of the interventions and investigate the measured outcomes including:
  a. Perinatal IPV, including revictimization and ongoing abuse;
  b. Adverse obstetrical and neonatal outcomes.
3. Identify knowledge gaps in the literature evaluating interventions for perinatal IPV.

The protocol for this review is published in *BMJ Open* (Fu et al., 2023). The protocol was developed in accordance with the Preferred Reporting Items for Scoping Reviews (PRISMA‐ScR) guidelines and the Joanna Briggs Institute methodology for scoping reviews (Peters et al., 2020; Tricco et al., 2018). There are no differences between the protocol and the review.

## 4 METHODS

### 4.1 Criteria for considering studies for this review

#### 4.1.1 Types of studies

All study designs reporting primary data on an intervention designed to target IPV during the perinatal period were included. This includes all study designs, such as RCTs, cohort studies, case control studies, pre‐test post‐test designs, qualitative studies, and mixed methods studies. Articles were included regardless of country of origin, however only articles written in English and French were included due to lack of resources to translate articles. All included studies were peer‐reviewed.

#### 4.1.2 Types of participants

Eligible participants were women (and others carrying a pregnancy) experiencing IPV during the perinatal period. There were no restrictions based on sexuality, race, religion, or any other sociodemographic factors. All participants in included studies were screened for IPV before study enrollment, as only secondary IPV prevention interventions were included in this review.

#### 4.1.3 Types of interventions

All interventions that targeted IPV during the perinatal period are included in this review. IPV screening programs or interventions evaluating primary prevention for IPV were excluded because the aim of this review is to describe interventions designed for those who are currently experiencing violence. Intervention types include cash transfer programs, home visitation programs, education‐based interventions, and counseling‐based interventions. Interventions designed to reduce or prevent IPV revictimization, or any adverse maternal, obstetrical, or neonatal health outcomes related to IPV victimization were included. Additionally, interventions designed to target a wide variety of outcomes related to family health, such as poverty, childhood growth, etc. that also reported on IPV revictimization and associated outcomes were included.

Included studies were targeted at women experiencing IPV during the perinatal period. Interventions solely targeting the providers involved in the care of perinatal women (e.g., provider training to increase referrals to community resources) were excluded. In the case where an intervention was targeted at both the provider and IPV survivors (e.g., provider training and assisted referrals to community resources), only the component of the intervention targeted at the IPV survivors is described in this review. Additionally, included interventions were targeted at the woman experiencing IPV or at the woman experiencing IPV and other family members (e.g., the perpetrator, children). Interventions targeting only the perpetrator or child were excluded.

Any intervention designed solely to increase IPV screening and identification was excluded because universal screening itself does not reduce adverse outcomes associated with IPV. Additionally, any intervention that too closely resembled normal prenatal care was excluded, as defined by the individual studies to reflect their typical care practices. For example, interventions composed of a brochure describing IPV and providing a list of local resources were considered standard of care and were excluded.

#### 4.1.4 Types of outcome measures

##### Primary outcomes

Studies were included in this review if they reported on any outcome related to adverse effects of IPV victimization during the perinatal period. This includes outcomes related to:

- Revictimization of IPV (e.g., physical, psychological, sexual)
- Maternal physical health (e.g., physical functioning, maternal nutrition)
- Maternal mental health (e.g., depression, PTSD, substance use)
- Obstetrical health (e.g., late entry into prenatal care, gestational diabetes, miscarriage, stillbirth)
- Neonatal health (e.g., neonatal intensive care unit admission)
- Parenting and family dynamics (e.g., parenting competence, child abuse)
- Knowledge of IPV and actions to reduce violence (e.g., safety planning, use of community resources)
- Intervention acceptability and feasibility

##### Secondary outcomes

No secondary outcomes were included in the review.

#### 4.1.5 Duration of follow‐up

Not applicable to this study.

#### 4.1.6 Types of settings

Interventions for perinatal IPV frequently occur during prenatal care, which often takes place in‐hospital, in hospital‐based clinics, and in community‐based clinics. Additionally, many home visitation programs have been designed to target perinatal IPV, meaning the intervention takes place in the participants' home. As well, many community‐based interventions have been designed to target perinatal IPV, ranging from cash transfer to counseling or education‐based interventions, and occur in community centers.

### 4.2 Search methods for identification of studies

#### 4.2.1 Electronic searches

Search strategies were developed by an information specialist (KF) and peer reviewed using the Peer Review of Electronic Search Strategies (PRESS) guideline (McGowan et al., 2016). The search encompassed January 2000 to June 2022 to retrieve most current peer‐reviewed articles, and the keywords and search strategy are included in the Supporting Information: Appendix. The search was run on June 1st, 2022, and the following electronic databases were searched:

- MEDLINE(R) ALL (OvidSP)
- Embase (OvidSP)
- CINAHL (EBSCOHost)
- APA PsycInfo (OvidSP)
- Cochrane Central Register of Controlled Trials (OvidSP)
- Web of Science
- Applied Social Sciences Index & Abstracts (ProQuest)

Each database was searched for the concept of “perinatal” and “IPV” using a combination of subject headings and keywords. Drafting the search strategy was informed by two Cochrane reviews for the concept of “IPV” (Hameed et al., 2020; Jahanfar et al., 2014), and by consulting the search method from the Cochrane Pregnancy and Childbirth's Trials Register (Cochrane Pregnancy and Childbirth Trials Register, 2022) for the concept of “perinatal.” No search filters or language limits were used. Non‐peer‐reviewed articles (e.g., commentaries, editorials) and conference abstracts were removed, since only full research articles were to be included in the review.

#### 4.2.2 Searching other resources

Additional sources were also searched, including ClinicalTrials.gov and MedRxiv. Hand searching of references from select articles was also performed.

### 4.3 Data collection and analysis

#### 4.3.1 Selection of studies

##### Title and abstract screening

Records retrieved by the search were uploaded to Covidence (*Covidence – Better Systematic Review Management*, 2023). The following exclusion criteria were used:

- Duplicate articles
- Non‐peer reviewed articles (e.g., conference abstracts)
- Not primary data collection
- Articles in a language other than English or French
- Study does not relate to IPV
- Study does not evaluate an intervention

Each title and abstract was screened by two independent reviewers (OM, YF, RF, AL) to ensure robustness. Conflicts were resolved by a third independent reviewer (KM).

##### Full‐text eligibility screening

All articles that passed through the title and abstract screening stage were screened according to the following exclusion criteria:

- Duplicate articles
- Non‐peer reviewed articles (e.g., conference abstracts)
- Not primary data collection
- Articles in a language other than English or French
- Study does not relate to IPV
- No/ineligible intervention (e.g., intervention does not target IPV, intervention targeted at providers only, primary IPV prevention)
- Ineligible participants (e.g., women outside of the perinatal period)

Each full‐text was screened by two independent reviewers (OM, YF, RF, AL). If a conflict arose, it was discussed with KM, and a consensus was achieved.

#### 4.3.2 Data extraction and management

Data were extracted from retained studies by a single reviewer (OM, YF, RF, AL). Data extraction was then confirmed by OM. Corresponding data were extracted in the following categories:

- General information including author name, publication year, journal of publication, country setting, study design, and sample size
- Population characteristics of the target of the intervention including method of IPV screening and referral process
- Intervention characteristics such as setting and type
- Measured outcomes
- Declarations involving funding, conflicts of interest, and ethics approval

The data extraction form was developed with guidance from the Template for Intervention Description and Replication (TIDieR) (Page et al., 2021). Data were summarized with descriptive statistics and narrative summary.

#### 4.3.3 Assessment of risk of bias in included studies

We did not assess risk of bias in included studies. This is a scoping review which does not synthesize effects of interventions and as per the PRISMA‐ScR guidelines and the Joanna Briggs Institute methodology for scoping reviews, assessment of risk of bias is not mandatory in scoping reviews if intervention effects are not synthesized (Peters et al., 2020; Tricco et al., 2018).

#### 4.3.4 Measures of treatment effect

Due to this being a scoping review, measures of treatment effect were not extracted or reported.

#### 4.3.5 Unit of analysis issues

We did not quantitatively synthesize effects across studies, but we did identify studies that used a cluster randomized design which is reported in the Characteristics of Included Studies table.

#### 4.3.6 Criteria for determination of independent findings

We ensured that we did not include multiple reports of the same study. When the same intervention was described in multiple studies, we made this clarification.

#### 4.3.7 Dealing with missing data

Due to this being a scoping review, we did not quantitatively synthesize effects, thus did not assess missing data. In studies that had missing data, we kept the study and did not include the missing information.

#### 4.3.8 Assessment of heterogeneity

Due to this being a scoping review, we did not quantitatively synthesize effects, thus did not assess heterogeneity.

#### 4.3.9 Assessment of reporting biases

Due to this being a scoping review, we did not assess reporting bias.

#### 4.3.10 Data synthesis

Data synthesis is narrative, and data were summarized in tables. For each included study, study design, country of conduct, number of participants, study population, comparator condition, intervention type(s), intervention setting and modality, and intervention descriptions are described. Additionally, the outcomes measured in included studies are summarized in both table and figure format.

#### 4.3.11 Subgroup analysis and investigation of heterogeneity

Due to this being a scoping review, we did not quantitatively synthesize effects, thus did not conduct subgroup analyses or investigation of heterogeneity.

#### 4.3.12 Sensitivity analysis

Due to this being a scoping review, we did not quantitatively synthesize effects, thus did not conduct sensitivity analyses.

#### 4.3.13 Treatment of qualitative research

Qualitative research was treated in the same manner as quantitative research. Interventions evaluated and findings were described using narrative synthesis, and data were summarized in tables.

## 5 RESULTS

### 5.1 Results of the search

The main search, completed in June 2022, yielded 23,265 records. One additional study was added via manual searching. After 13,173 duplicate records were removed, 10,093 were advanced to full title and abstract screening. Another 9,867 records were removed during this step, with 226 proceeding to the full text review. Of these, 67 met the inclusion criteria. Three records could not be retrieved due to lack of response from the authors.

Of the included studies, some studies are reported in multiple articles. The LEAP 1000 home visitation program was described in two articles (Barrington et al., 2022; Peterman et al., 2022); a trauma and violence‐informed cognitive behavioral therapy intervention was described in two articles (Jackson et al., 2020; Mantler et al., 2022); a psychosocial intervention in Nepal was described in two articles (Sapkota et al., 2020, 2022); the Safe Pregnancy Study was described in two articles (Flaathen et al., 2022; Walter et al., 2021); DOVE is described in three articles (Bacchus et al., 2016a, 2016b; Sharps et al., 2016); the Early Start Program is described in two articles (Fergusson et al., 2005, 2013); a professional home visitation program was described in three articles (Olds et al., 2004, 2007, 2010); and a home visitation program based in the United Kingdom was described in two articles (Barlow et al., 2007; McIntosh et al., 2009). Therefore, 57 unique interventions are described in this scoping review.

**Figure 1:**
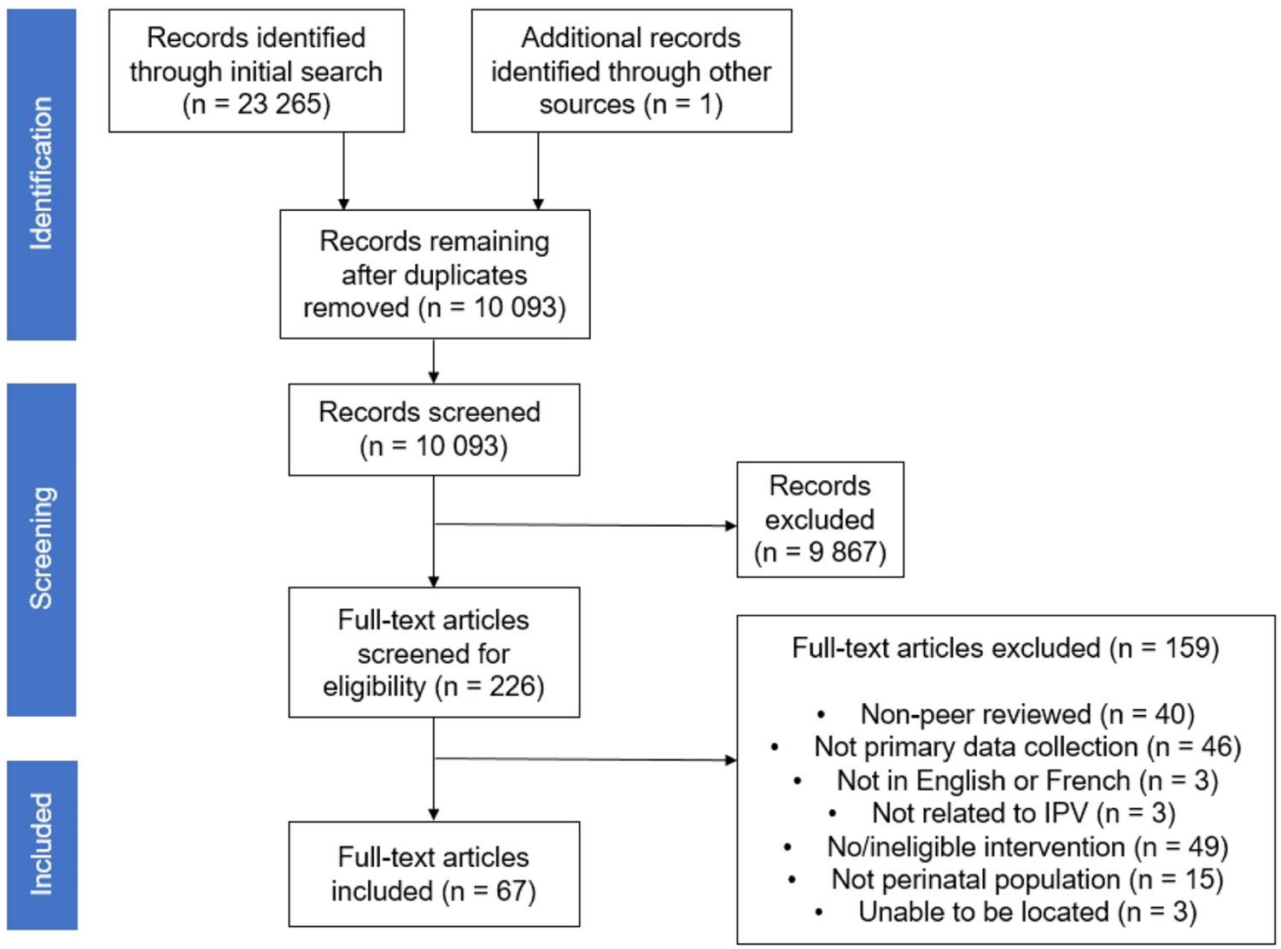
PRISMA diagram.

The search results are shown in the PRISMA diagram (Page et al., 2021) provided in Figure 1.

### 5.2 Excluded studies

A total of 226 full‐text articles were screened for eligibility, and of these, 159 articles were excluded (Figure 1). Articles were excluded because they were not peer‐reviewed (*n* = 40), did not describe primary data collection (*n* = 46), or were not published in English or French (*n* = 3). Articles were also excluded because they were not related to IPV (*n* = 3), did not evaluate an intervention or evaluated an ineligible intervention (i.e., intervention was targeted at the providers only) (*n* = 49), or participants were not in the perinatal period (*n* = 15). Finally, three articles could not be located.

### 5.3 Included studies

There are 67 studies included in this scoping review. Included studies were published between 2000 and 2022. An overview of the main characteristics of the included studies such as study design, country of conduct, number of participants, study population, intervention type, and comparator are provided in Table 1. While it is possible that these studies included trans men or non‐binary individuals, none of the included studies noted this. Therefore, we will use the term women through the results section.

**Table 1:** Characteristics of included studies.

Study title	References	Study design	Country of conduct	Number of participants	Study population at recruitment	Intervention type(s)	Comparator
*Cash Transfer*							
Poverty can break a home: exploring mechanisms linking cash plus programming and intimate partner violence in Ghana^a^	Barrington (2022)	Qualitative Study	Ghana	30	Women who are pregnant or have a child under the age of 12 months and who live in households that meet poverty‐related criteria.	Cash Transfer	No comparator.
Government Antipoverty Programming and Intimate Partner Violence in Ghana^a^	Peterman (2022)	Quasi‐Experimental study	Ghana	2331	Women who are pregnant or have a child under the age of 12 months and who live in households that meet poverty‐related criteria.	Cash Transfer	No comparator.
Evaluation of an unconditional cash transfer program targeting children's first‐1000‐days linear growth in rural Togo: A cluster‐randomized controlled trial	Briaux (2020)	Randomized Controlled Trial^d^	Togo	2658^b^	Antenatal and post‐partum women and their children living in rural landlocked villages with poor access to health facilities.	Cash Transfer; Education	Package of community activities including sensitization meetings and home visits directed at child health, nutrition, and protection, and integrated community care management of childhood illnesses and acute malnutrition targeted at mother‐child pairs during the first 1000 days.
*Counseling*							
Implementation outcomes of a health systems strengthening intervention for perinatal women with common mental disorders and experiences of domestic violence in South Africa: Pilot feasibility and acceptability study	Abrahams (2022)	Mixed Methods (Pre‐Test Post‐Test and Qualitative)	South Africa	139	Antenatal women with mixed‐ancestry, Black African women, or Black South African women.	Counseling	No comparator.
The effect of family‐based counseling on domestic violence in pregnant women referring to health centers in Sahneh city, Iran, 2018	Babaheidarian (2021)	Randomized Controlled Trial	Iran	92	Antenatal women exposed to moderate to high violence that were referred to a psychiatrist to confirm violence.	Counseling	Routine prenatal care and an educational package. A training package was provided following the study. Participants were visited by a psychiatrist during and after the study to identify possible complications and risks.
Evaluation of a domestic violence intervention in the maternity and sexual health services of a UK hospital	Bacchus (2010)	Qualitative Study	United Kingdom	1131	Antenatal women attending maternal or sexual health clinics.	Counseling	No comparator.
The effectiveness of empowerment program on increasing self‐esteem, learned resourcefulness, and coping ways in women exposed to domestic violence	Bahadir‐Yilmaz (2018)	Randomized Controlled Trial^c^	Turkey	60	Antenatal and post‐partum women who were literate.	Counseling	No intervention for control group.
Exploring the impact of a community participatory intervention on women's capability: a qualitative study in Gulu Northern Uganda	Belaid (2021)	Qualitative Study	Uganda	14	Antenatal and post‐partum women.	Counseling	No comparator.
For Baby's Sake: Intervention Development and Evaluation Design of a Whole‐Family Perinatal Intervention to Break the Cycle of Domestic Abuse	Domoney (2019)	Mixed Methods (Surveys and Qualitative)	England	40	Antenatal and post‐partum women where the father is the main perpetrator of IPV.	Counseling	No comparator.
An intervention to improve post‐partum outcomes in African American mothers: a randomized controlled trial	El‐Mohandes (2008)	Randomized Controlled Trial	United States of America	913	Antenatal English speaking African American women with minority status living in DC (Washington).	Counseling	Usual prenatal care, defined as meeting with their primary care providers as per standard clinic practice.
The Young Parenthood Program: Preventing Intimate Partner Violence Between Adolescent Mothers and Young Fathers	Florsheim (2011)	Randomized Controlled Trial	United States of America	105	Antenatal adolescents considered at‐risk and the biological father of the child. First‐time mothers had to be between 14 and 18 years old; biological fathers had to be between 14 and 24 years old.	Counseling	Prenatal services and access to some psychosocial services, (i.e., vocational counseling and parenting classes).
Addressing intimate partner violence and power in intimate relationships in HIV testing services in Nairobi, Kenya	Haberland (2020)	Randomized Controlled Trial	Kenya	698	First‐time antenatal care clinic patients.	Counseling	Standard HIV testing services.
Promoting attachment through healing (PATH): results of a retrospective feasibility study providing trauma‐and‐violence‐informed care to pregnant women^a^	Mantler (2022)	Retrospective Case Control Study	Canada	69	Antenatal and post‐partum women with depression, anxiety, or PTSD.	Counseling	Referral to a psychiatrist, social worker and/or a family physician for further treatment and support.
Exploring mothers' experiences of trauma and violence‐informed cognitive behavioral therapy following intimate partner violence: a qualitative case analysis^a^	Jackson (2020)	Qualitative Study	Canada	3	English‐speaking post‐partum Caucasian women.	Counseling	No comparator.
Preventing intimate partner violence among teen mothers: a pilot study	Kan (2021)	Randomized Controlled Trial	United States of America	32	Pregnant and parenting teens.	Counseling	Original Safe Dates curriculum which included interactive and engaging skills‐building opportunities for teens, including modeling of skills by implementers and opportunities for teens to practice and receive feedback on skills.
The design, implementation, and acceptability of an integrated intervention to address multiple behavioral and psychosocial risk factors among pregnant African American women	Katz (2008)	Mixed Methods (Pre‐Test Post‐Test and Qualitative)	United States of America	216	African American, minority women of low SES, living in an urban setting.	Counseling	Standard prenatal care.
Screening and brief intervention for intimate partner violence among antenatal care attendees at primary healthcare clinics in Mpumalanga province, South Africa	Matseke (2013)	Pre‐Test Post‐Test Design	South Africa	160	Antenatal women.	Counseling	No comparator.
Group problem solving therapy for perinatal depression in primary health care settings in rural Uganda: an intervention cohort study	Nakku (2021)	Prospective Cohort Study	Uganda	153	Antenatal women in their second or third trimester living in a rural Uganda, who spoke English or Luganda and had depression (as determined by a midwife).	Counseling	No comparator.
An Integrated Randomized Intervention to Reduce Behavioral and Psychosocial Risks: Pregnancy and Neonatal Outcomes	Subramanian (2012)	Randomized Controlled Trial	United States of America	1025	Antenatal low‐income urban African American women.	Counseling	Usual care.
A randomized controlled trial empowerment training for Chinese abused pregnant women in Hong Kong	Tiwari (2005)	Randomized Controlled Trial	Hong Kong	110	Antenatal Chinese women.	Counseling	Standard care for abused women, consisting of a wallet‐sized card with information on community resources for abused women (shelter hotlines, law enforcement, social services and non‐government organizations).
A brief motivational intervention to address intimate partner violence victimization: A pilot study	Trabold (2020)	Mixed Methods (Pre‐Test Post‐Test and Qualitative)	United States of America	6	Antenatal and post‐partum women able to understand English, 83% Caucasian, majority of patients met federal poverty guidelines.	Counseling	No comparator.
An interpersonally based intervention for low‐income pregnant women with intimate partner violence: a pilot study	Zlotnick (2011)	Randomized Controlled Trial	United States of America	54	Antenatal low‐income women.	Counseling	Usual antenatal medical care, the educational material, and a listing of resources for IPV.
*Education*							
The effect of sexual health education on sexual activity, sexual quality of life, and sexual violence in pregnancy: a prospective randomized controlled trial	Alizadeh (2021)	Randomized Controlled Trial	Iran	225	Antenatal women, 53% of participants were African American.	Education	Routine care and no sex education.
Safe Pregnancy intervention for intimate partner violence: a randomized controlled trial in Norway among culturally diverse pregnant women^a^	Flaathen (2022)	Randomized Controlled Trial	Norway	317	Antenatal women.	Education	Received general information about different aspects of having a healthy and safe pregnancy, recommendations regarding a healthy diet, exercise, alcohol consumption, and smoking and brief information about where to get help if exposed to IPV.
Pregnant Women's Attitudes Toward and Experiences With a Tablet Intervention to Promote Safety Behaviors in a Randomized Controlled Trial: Qualitative Study^a^	Walter (2021)	Qualitative Study	Norway	10	Antenatal women.	Education	Women watched a film with general information about health during pregnancy, including information about where to find help if experiencing violence.
Impacts of a coparenting‐focused intervention on links between pre‐birth intimate partner violence and observed parenting	Kan (2015)	Randomized Controlled Trial	United States of America	169	Antenatal women.	Education	Received a brochure by mail providing information on selecting quality childcare.
An integrated intervention to reduce intimate partner violence in pregnancy: A randomized trial	Kiely (2010)	Randomized Controlled Trial	United States of America	1044	Antenatal African American women.	Education	Normal prenatal care.
A family planning clinic partner violence intervention to reduce risk associated with reproductive coercion	Miller (2011)	Randomized Controlled Trial	United States of America	1207	Antenatal women who were mostly non‐Caucasian in an urban setting.	Education	Standard of care, which involves responding to two violence screening questions on an intake form. In the event of a positive disclosure in the control clinics, RHS and clinicians follow a standard clinic protocol, including filing any necessary mandated reports, documenting IPV on the client chart and giving the client a list of violence victimization resources.
Improving Safety Among Pregnant Women Reporting Domestic Violence in Nepal‐A Pilot Study	Rishal (2020)	Pre‐Test Post‐Test Design	Nepal	80	Antenatal women.	Education	No comparator.
Maternal and child health nurse screening and care for mothers experiencing domestic violence (MOVE): a cluster randomized trial	Taft (2015)	Randomized Controlled Trial^d^	Australia	163	Low‐income post‐partum women in an urban setting.	Education	Usual care involved government mandated face‐to‐face domestic violence screening at 4 weeks post‐partum and follow‐up as required.
The effect of training problem‐solving skills for pregnant women experiencing intimate partner violence: a randomized control trial	Taghizadeh (2018)	Randomized Controlled Trial	Iran	284	Antenatal women.	Education	Normal prenatal care.
A community‐supported clinic‐based program for prevention of violence against pregnant women in rural Kenya	Turan (2013)	Mixed Methods (Surveys, Focus Groups, Interviews)	Kenya	49	Antenatal African women in a rural setting.	Education	No comparator.
The impact of a referral card‐based intervention on intimate partner violence, psychosocial health, help‐seeking and safety behavior during pregnancy and post‐partum: a randomized controlled trial	Van Parys (2017)	Randomized Controlled Trial	Belgium	249	Post‐partum women.	Education	Received a thank you card.
*Counseling and Education*							
The Impact of Preventive Interventions on Intimate Partner Violence among Pregnant Women Resident in Hamadan City Slum Areas Using the PEN‐3 Model: Control Randomized Trial Study	Rastegar (2021)	Randomized Controlled Trial	Iran	150	Low‐income antenatal women in an urban setting.	Counseling; Education	Standard antenatal care.
Effect of counseling on the family function of intimate partner violence victims attending antenatal Central Nigeria clinic in a tertiary hospital in North	Akor (2019)	Randomized Controlled Trial	Nigeria	72	Antenatal women <34 weeks' gestation.	Counseling; Education	Normal antenatal care.
Effectiveness of a counseling intervention implemented in antenatal setting for pregnant women facing domestic violence: a preexperimental study	Arora (2019)	Pre‐Test Post‐Test Design	India	155	Antenatal women in Mumbai.	Counseling; Education	No comparator.
Intimate partner violence during pregnancy: a pilot intervention program in Lima, Peru	Cripe (2010)	Randomized Controlled Trial	Peru	220	Antenatal women that spoke Spanish.	Counseling; Education	Wallet‐size referral card listing agencies that provided IPV services to abused women.
Nurse case management for pregnant women experiencing or at risk for abuse	Curry (2006)	Randomized Controlled Trial	United States of America	1000	Antenatal women that spoke English. Intentional inclusion of 20 adolescents due to higher risk of abuse.	Counseling; Education	Small trifold card with safety and abuse recognition information that included telephone numbers for local and national domestic violence resources.
The effect of solution focused counseling on violence rate and quality of life of pregnant women at risk of domestic violence: a randomized controlled trial	Dinmohammadi (2021)	Randomized Controlled Trial	Iran	90	Antenatal women in an urban center.	Counseling; Education	Governmental childbirth preparation classes teaching pregnancy, breastfeeding, and other exercises.
The effect of a supportive‐educational intervention on maternal‐fetal attachment of pregnant women facing domestic violence: A randomized controlled trial	Khalili (2020)	Randomized Controlled Trial	Iran	100	Antenatal women.	Counseling; Education	Routine prenatal care.
Safe mom, safe baby: a collaborative model of care for pregnant women experiencing intimate partner violence	Kramer (2012)	Pre‐Test Post‐Test Design	United States of America	340	Antenatal and post‐partum women; majority were non‐white women with limited economic resources in a large city.	Counseling; Education	No comparator.
A brief intervention for prevention of sexually transmitted infection among battered women	Laughon (2011)	Pre‐Test Post‐Test Design	United States of America	18	Antenatal women living in a rural setting.	Counseling; Education	No comparator.
An evaluation of interventions to decrease intimate partner violence to pregnant women	McFarlane (2000)	Randomized Controlled Trial	United States of America	329	Antenatal women; predominantly Hispanic women in an urban center.	Counseling; Education	No comparator.
A family planning clinic‐based intervention to address reproductive coercion: A cluster randomized controlled trial	Miller (2016)	Randomized Controlled Trial^d^	United States of America	4009	Antenatal English and Spanish speaking women.	Counseling; Education	Usual care including a standard IPV question on intake sheet and provision of referral if IPV disclosed. Offered a women's health resource sheet.
A psychosocial intervention to reduce gender‐based violence and antepartum depressive symptoms in pregnant women in Kisumu County, Kenya: A quasi‐experimental study	Mutisya (2018)	Quasi‐Experimental study	Kenya	288	Antenatal women.	Counseling; Education	Usual antenatal care services and a card listing local persons/organizations where help for gender‐based violence could be sought. Received one psychosocial session with the research assistant after the final interview.
A quasi‐experimental outcomes analysis of a psychoeducation intervention for pregnant women with abuse‐related posttraumatic stress	Rowe (2014)	Quasi‐Experimental study	United States of America	60	Antenatal English‐speaking women.	Counseling; Education	The comparison used were results from a prospective observational study where women received usual care. In the study, women were divided into three groups: lifetime PTSD cases, trauma‐exposed (resilient) controls, and non‐exposed controls. The study included structured psychiatric diagnostic interviews conducted by telephone.
Antenatal‐Based Pilot Psychosocial Intervention to Enhance Mental Health of Pregnant Women Experiencing Domestic and Family Violence in Nepal^a^	Sapkota (2022)	Randomized Controlled Trial	Nepal	140	Antenatal women of African American, European American, Latina, Asian, or Native American ethnicity.	Counseling; Education	Usual antenatal care and a booklet including a referral list of local support organizations working against domestic and family violence.
“We don't see because we don't ask”: Qualitative exploration of service users' and health professionals' views regarding a psychosocial intervention targeting pregnant women experiencing domestic and family violence^a^	Sapkota (2020)	Qualitative Study	Nepal	63	Post‐partum women.	Counseling; Education	No comparator.
A Randomized Controlled Trial of a Computer‐Based Brief Intervention for Victimized Perinatal Women Seeking Mental Health Treatment	Zlotnick (2019)	Randomized Controlled Trial	United States of America	53	Pregnant or within 6‐months post‐partum women seeking mental health treatment at an urban health clinic.	Counseling; Education	Control group interacted with the computer software, but content showed brief segments of popular television shows and asked for their ratings.
*Home Visitation*							
Randomized trial of the early start program of home visitation^a^	Fergusson (2005)	Randomized Controlled Trial	New Zealand	443	Low‐income antenatal and post‐partum women.	Home Visitation	No intervention.
Nine‐year follow‐up of a home‐visitation program: a randomized trial^a^	Fergusson (2013)	Randomized Controlled Trial	New Zealand	443	Low‐income antenatal and post‐partum women.	Home Visitation	No intervention.
Randomized trial of a statewide home visiting program to prevent child abuse: impact in reducing parental risk factors	Duggan (2004)	Randomized Controlled Trial	United States of America	643	Post‐partum women.	Home Visitation	No intervention.
Effects of Home Visits by Paraprofessionals and by Nurses: Age 4 Follow‐Up Results of a Randomized Trial^a^	Olds (2004)	Randomized Controlled Trial	United States of America	695	Antepartum low‐income women who had no previous live births and either qualified for Medicaid or had no private insurance.	Home Visitation	Free developmental screening and referral for their children at 6, 12, 15, 21, and 24 months of age.
Effects of Nurse Home Visiting on Maternal and Child Functioning: Age‐9 Follow‐up of a randomized Trial^a^	Olds (2007)	Randomized Controlled Trial	United States of America	743	Antenatal women <29 weeks' gestation with no previous live births and at least 2 sociodemographic risk characteristics (unmarried, <12 years of education, unemployed). Primarily African American women.	Home Visitation	Control 1: free round‐trip taxicab transportation for scheduled prenatal care appointments. Control 2: free transportation for scheduled prenatal care plus developmental screening and referral services for the child at 6, 12, and 24 months of age.
Enduring Effects of Prenatal and Infancy Home Visiting by Nurses on Maternal Life Course and Government Spending: Follow‐up of a randomized controlled trial among children at age 12 years^a^	Olds (2010)	Randomized Controlled Trial	United States of America	594	Antenatal women <29 weeks' gestation with no previous live births and at least 2 sociodemographic risk characteristics (unmarried, <12 years of education, unemployed). Primarily African American women.	Home Visitation	Free transportation for scheduled prenatal care appointments plus developmental screening and referral services for the child at 6, 12, and 24 months of age.
Mothers' AdvocateS In the Community (MOSAIC)—non‐professional mentor support to reduce intimate partner violence and depression in mothers: a cluster randomized trial in primary care	Taft (2011)	Randomized Controlled Trial^d^	Australia	215	Antenatal and post‐partum women.	Home Visitation; Counseling; Education	Single home visitation session to negotiate consent and provide a resource card for new mothers which included contacts for family violence services.
Promoting parenting in home visiting: A CACE analysis of Family Foundations	Ammerman (2022)	Randomized Controlled Trial	United States of America	150	Antenatal and post‐partum women.	Home Visitation; Education	Home visitation that included early prevention, education of mothers, helping mothers achieve independence and economic self‐sufficiency, identifying home needs.
Role of home visiting in improving parenting and health in families at risk of abuse and neglect: results of a multicentre randomized controlled trial and economic evaluation^a^	Barlow (2007)	Randomized Controlled Trial	United Kingdom	131	Antenatal women.	Home Visitation; Education	Standard help currently available to such families.
Economic evaluation of an intensive home visiting program for vulnerable families: a cost‐effectiveness analysis of a public health intervention^a^	McIntosh (2009)	Randomized Controlled Trial	United Kingdom	131	Antenatal women.	Home Visitation; Education	Standard help currently available to such families.
An Intimate Partner Violence Prevention Intervention in a Nurse Home Visitation Program: A Randomized Clinical Trial	Feder (2018)	Randomized Controlled Trial	United States of America	330	Low‐income, first‐time pregnant women.	Home Visitation; Education	Participated In the Nurse Family Partnership Program with nurses who lacked special IPV training.
Effect of Addition of an Intimate Partner Violence Intervention to a Nurse Home Visitation Program on Maternal Quality of Life A Randomized Clinical Trial	Jack (2019)	Randomized Controlled Trial	United States of America	492	Socially disadvantaged antenatal women.	Home Visitation; Education	Standard nurse home visitation by nurses without specific IPV training.
Improving Adolescent Parenting: Results From a Randomized Controlled Trial of a Home Visiting Program for Young Families	Jacobs (2016)	Randomized Controlled Trial	United States of America	704	Antenatal and post‐partum women.	Home Visitation; Education	Information about child development and referrals to other services, but no Healthy Families Massachusetts services.
Effect of nurse home visits versus usual care on reducing intimate partner violence in young high‐risk pregnant women: a randomized controlled trial	Mejdoubi (2013)	Randomized Controlled Trial	Netherlands	460	Antenatal and post‐partum low‐educated women.	Home Visitation; Education	The usual care was provided consisting of maternal healthcare during pregnancy, home visits after birth, and check‐ups until the child's second birthday. Families with special needs could receive support from (child) welfare organizations and mental health services.
Domestic Violence Enhanced Perinatal Home Visits: The DOVE Randomized Clinical Trial^a^	Sharps (2016)	Randomized Controlled Trial	United States of America	108	Antenatal women.	Home Visitation; Education	Usual care of the home visitation program (4–6 visits prenatally and 6–12 visits up to 2 years post‐partum).
Infusing Technology Into Perinatal Home Visitation in the United States for Women Experiencing Intimate Partner Violence: Exploring the Interpretive Flexibility of an mHealth Intervention^a^	Bacchus (2016)	Qualitative Study	United States of America	26	Antenatal women.	Home Visitation; Education	No comparator.
“Opening the door”: a qualitative interpretive study of women's experiences of being asked about intimate partner violence and receiving an intervention during perinatal home visits in rural and urban settings in the USA^a^	Bacchus (2016)	Qualitative Study	United States of America	26	Antenatal women.	Home Visitation; Education	No comparator.
Assessing suitability for a couple‐based intervention for domestic abuse: learning from a feasibility study	McConnell (2020)	Mixed Methods (Surveys and Interviews)	United Kingdom	70	Antenatal and post‐partum women.	Home Visitation; Counseling; Education	No comparator.

a Studies were conducted to evaluate the same intervention.

b Number of participants includes women experiencing IPV and their children.

c RCT with pre‐ and post‐test comparison.

d Cluster RCT.

### 5.4 Description of studies

#### 5.4.1 Study location

Included studies were conducted in 19 different countries around the world. The majority were conducted in North America with 29 conducted in the United States (Ammerman et al., 2022; Bacchus et al., 2016a, 2016b; Curry et al., 2006; Duggan et al., 2004; El‐Mohandes et al., 2008; Feder et al., 2018; Florsheim et al., 2011; Jack et al., 2019; Jacobs et al., 2016; Kan & Feinberg, 2015; Kan et al., 2021; Katz et al., 2008; Kiely et al., 2010; Kramer et al., 2012; Laughon et al., 2011; McFarlane et al., 2000; Miller et al., 2011, 2016; Olds et al., 2004, 2007, 2010; Peterman et al., 2022; Rowe et al., 2014; Sharps et al., 2016; Subramanian et al., 2012; Trabold et al., 2020; Zlotnick et al., 2011, 2019) and two conducted in Canada (Jackson et al., 2020; Mantler et al., 2022). A total of 5 studies were conducted in the United Kingdom or England (Bacchus et al., 2010; Barlow et al., 2007; Domoney et al., 2019; McConnell et al., 2020; McIntosh et al., 2009). A total of four studies were conducted in Oceania, two in Australia (Taft et al., 2011, 2015) and two and New Zealand (Fergusson et al., 2005, 2013). Meanwhile, some included studies were conducted in African countries, including South Africa (Abrahams et al., 2022; Matseke & Peltzer, 2013), Nigeria (Akor et al., 2019), Ghana (Barrington et al., 2022), Togo (Briaux et al., 2020), Kenya (Haberland et al., 2020; Mutisya et al., 2018; Turan et al., 2013), and Uganda (Belaid et al., 2021; Nakku et al., 2021). Multiple studies were conducted in Asian and Middle Eastern countries, including Iran (Alizadeh et al., 2021; Babaheidarian et al., 2021; Dinmohammadi et al., 2021; Khalili et al., 2020; Rastegar et al., 2021; Taghizadeh et al., 2018), Nepal (Rishal et al., 2020; Sapkota et al., 2020, 2022), Hong Kong (Tiwari et al., 2005), India (Arora et al., 2019), and Turkey (Bahadir‐Yilmaz & Öz, 2018). A total of 4 studies were conducted in European countries, including Norway (Flaathen et al., 2022; Walter et al., 2021), Belgium (Van Parys et al., 2017), and the Netherlands (Mejdoubi et al., 2013). Only one included study was conducted in South America, and was conducted in Peru (Cripe et al., 2010).

#### 5.4.2 Study design characteristics

##### Study design

The majority (*n* = 43) of included studies were RCTs. Six studies utilized a mixed methods design, using a combination of pre‐test post‐test surveys, focus groups, and/or interviews (Abrahams et al., 2022; Domoney et al., 2019; Katz et al., 2008; McConnell et al., 2020; Trabold et al., 2020; Turan et al., 2013). Five studies used single‐group pre‐post intervention designs (Arora et al., 2019; Kramer et al., 2012; Laughon et al., 2011; Matseke & Peltzer, 2013; Rishal et al., 2020). Three studies used a quasi‐experimental design (Mutisya et al., 2018; Peterman et al., 2022; Rowe et al., 2014), one study utilized a prospective cohort study design (Nakku et al., 2021), one study utilized a retrospective case control study design (Mantler et al., 2022), and eight studies took a solely qualitative approach to evaluating study outcomes (Bacchus et al., 2010, 2016a, 2016b; Barrington et al., 2022; Belaid et al., 2021; Jackson et al., 2020; Sapkota et al., 2020; Walter et al., 2021).

##### Comparators

In many studies (*n* = 12), participants in the control group received normal prenatal care (Akor et al., 2019; Alizadeh et al., 2021; Barlow et al., 2007; El‐Mohandes et al., 2008; Haberland et al., 2020; Katz et al., 2008; Khalili et al., 2020; Kiely et al., 2010; McIntosh et al., 2009; Rastegar et al., 2021; Subramanian et al., 2012; Taghizadeh et al., 2018). Additionally, in two studies, normal prenatal care included further follow‐up upon positive screening of IPV according to clinic standards (Miller et al., 2011; Taft et al., 2015).

Meanwhile, in most studies (*n* = 26), individuals in the control group were provided with supports additional those that would be provided in normal prenatal or post‐partum care. Six studies provided a resource card with information about community organizations that provide care for IPV survivors (Cripe et al., 2010; Curry et al., 2006; Miller et al., 2016; Sapkota et al., 2022; Tiwari et al., 2005; Zlotnick et al., 2011). Two studies provided education on how to be healthy during pregnancy as well as brief information about where to get help if exposed to IPV (Flaathen et al., 2022; Walter et al., 2021) and one study provided a single home visitation session to provide a resource card with community organization information (Taft et al., 2011). Meanwhile, some studies provided additional services such as referral to a psychiatrist or other mental health services (Babaheidarian et al., 2021; Mantler et al., 2022; Mejdoubi et al., 2013; Mutisya et al., 2018; Rowe et al., 2014). Some studies provided resources and education unrelated to IPV such as breastfeeding classes (Dinmohammadi et al., 2021), parenting classes (Florsheim et al., 2011), community activities and education (Briaux et al., 2020), and developmental screening for children (Olds et al., 2004, 2007, 2010). In some studies, the control group received the same resources as the intervention group, except the information given to the control group was not tailored to IPV. Four studies provided the usual home visitation services (Ammerman et al., 2022; Feder et al., 2018; Jack et al., 2019; Sharps et al., 2016), while Kan et al. provided the original Safe Dates curriculum (i.e., not catered to IPV) (Kan et al., 2021). Finally, in three studies, participants in the control group were provided with unrelated services including a brochure about selecting quality childcare (Kan & Feinberg, 2015), a thank you card (Van Parys et al., 2017), and a video related to television shows (Zlotnick et al., 2019).

Of the 67 studies, three provided no resources or support to women in the control group (Duggan et al., 2004; Fergusson et al., 2005, 2013). Additionally, a total of 20 studies reported no comparator condition. Of these, five are pre‐test post‐test designs (Arora et al., 2019; Kramer et al., 2012; Laughon et al., 2011; Matseke & Peltzer, 2013; Rishal et al., 2020), one used a prospective cohort study design (Nakku et al., 2021), and one used a quasi‐experimental study design (Peterman et al., 2022). Additionally, five studies did not include a comparison group as they were pilot studies evaluating initial acceptability and feasibility of the intervention (Abrahams et al., 2022; Domoney et al., 2019; McConnell et al., 2020; Trabold et al., 2020; Turan et al., 2013). Seven qualitative studies (Bacchus et al., 2010, 2016a, 2016b; Barrington et al., 2022; Belaid et al., 2021; Jackson et al., 2020; Sapkota et al., 2020) did not include a comparison group, and one RCT included three intervention groups rather than a comparison group (McFarlane et al., 2000).

#### 5.4.3 Participant characteristics

The included studies included a total of 27,327 participants. Most studies included prenatal and/or post‐partum participants in the surrounding area who were experiencing IPV. However, many studies restricted their study population to only include women with certain sociodemographic factors. Sixteen studies included only women of low socioeconomic status or other associated factors (i.e., low education) (Barrington et al., 2022; Feder et al., 2018; Fergusson et al., 2005, 2013; Jack et al., 2019; Katz et al., 2008; Mejdoubi et al., 2013; Olds et al., 2004, 2007, 2010; Peterman et al., 2022; Rastegar et al., 2021; Subramanian et al., 2012; Taft et al., 2015; Trabold et al., 2020; Zlotnick et al., 2011). Meanwhile, four studies restricted their study to women living in rural settings (Briaux et al., 2020; Laughon et al., 2011; Nakku et al., 2021; Turan et al., 2013), and two included only adolescents (Florsheim et al., 2011; Kan et al., 2021). A total of five studies restricted study participants by ethnicity, including individuals of minority status only (El‐Mohandes et al., 2008), Caucasian Canadian women (Jackson et al., 2020), African American women (Kiely et al., 2010; Subramanian et al., 2012), and Hispanic women (McFarlane et al., 2000). Finally, four studies restricted their study population to only include women with pre‐existing mental health concerns (Babaheidarian et al., 2021; Mantler et al., 2022; Nakku et al., 2021; Zlotnick et al., 2019).

#### 5.4.4 Intervention characteristics

The key characteristics of included interventions are described in Table 2, including intervention type, intervention setting and modality, and detailed descriptions of each intervention.

**Table 2:** Description of interventions evaluated in included studies.

References	Intervention type(s)	Type of study	Package of intervention or IPV focus	Intervention setting and modality	Intervention description	Brief Intervention description
*Cash Transfer*						
Peterman (2022)^a^	Cash Transfer	Quasi‐experimental study	Package	Home; One‐on‐one	LEAP 1000 provides bimonthly unconditional cash payments (USD $10–$18) combined with premium health insurance under the National Health Insurance Authority. Fees related to card processing, premiums, and renewals were waived, however participants must take the initiative to enroll in the National Health Insurance Scheme.	Cash transfer combined with health insurance.
Barrington (2022)^a^	Cash Transfer	Qualitative Study	Package	Home; One‐on‐one	LEAP 1000 provides bimonthly unconditional cash payments (USD $10–$18) combined with premium health insurance under the National Health Insurance Authority. Fees related to card processing, premiums, and renewals were waived, however participants must take the initiative to enroll in the National Health Insurance Scheme.	Cash transfer combined with health insurance.
Briaux (2020)	Cash Transfer; Education	Randomized Controlled Trial^c^	Package	Home and community center; Group and one‐on‐one	Monthly cash distributions to women (US$8.30/month) combined with behavior change communication (BCC) activities (including home visits and community sensitization meetings) with the integrated community case management of childhood illnesses and acute malnutrition. BCC sessions focusing on child protection issues (e.g., birth registration, schooling, or fostering) were organized. Women were strongly encouraged to adopt specific behaviors conducive to their children's protection and well‐being, namely, to fulfill at least 4 antenatal visits, register children's births, enroll children in primary school, keep children younger than 15 at home (no fostering), and attend community health worker (CHW) and community child protection worker (CCPW) sensitization meetings around essential family practices. Women attending sensitization meetings received a bonus of US$33 when exiting the program.	Cash transfer combined with basic healthcare for children and pregnant women, education, and home visitation.
*Counseling*						
Abrahams (2022)	Counseling	Mixed Methods (Pre‐Test post‐test and Qualitative)	IPV Focus	Community clinic; One‐on‐one	Health Systems Strengthening program included provision of psychological counseling sessions to identified women. Healthcare workers received training to become equipped with knowledge and skills to detect common mental disorders and domestic violence and learned to delivery individual‐level care.	Program focused on providing counseling sessions to identified women.
Babaheidarian (2021)	Counseling	Randomized Controlled Trial	IPV Focus	Hospital; Group	Three 45‐min counseling sessions where pregnant women were asked to choose a member of their family to attend their counseling sessions, such as a spouse, mother, or mother‐in‐law. Counseling sessions were conducted according to Greeting, Ask, Tell, Help, Explain, and Refer (GATHER) principles. The content materials of the counseling were decided according to the information obtained from the questionnaire and the type of violence applied, along with considering the secrecy and safety issues of the family.	Counseling sessions that included one family member of pregnant woman's choosing.
Bacchus (2010)	Counseling	Qualitative Study	IPV Focus	Community clinic; One‐on‐one	Referral to an on‐site advocacy service that assists women accessing community resources and provides emotional support.	Patient referral to an advocacy service that provides emotional support and assistance in accessing community resources.
Bahadir‐Yilmaz (2018)	Counseling	Randomized Controlled Trial^b^	IPV Focus	Hospital and community clinic; One‐on‐one	Empowerment program based on individual counseling consisting of 10 sessions. The program was developed based on the subject. Each session lasted for 90 min once a week and focused on developing self‐awareness, increasing self‐esteem, decreasing learned helplessness, increasing learned resourcefulness, making sense of violence, managing violence, increasing effective coping, and using social resources to cope with violence.	Counseling session focused on empowering women.
Belaid (2021)	Counseling	Qualitative	Package	Community center; Group	Community‐based women's groups using a participatory learning and action (PLA) cycle, where a trained female facilitator discusses mothers' and childrens' health issues with women from her community. PLA approach integrated into pre‐existing savings groups (small groups where members pool their savings contributions and then borrow from them). Each group selected members to facilitate the meetings using the PLA cycle, and groups met weekly. The meetings followed a four‐phase cycle: (1) identify and prioritize problems that may occur during and after delivery and during childhood; (2) plan activities to reduce these problems; (3) implement strategies to address the priority problems; (4) assess the activities. In phase 3, facilitators invited men and other community members to discuss the implementation of the strategies to address the problems identified in phase 1. The project provided each group with US$30 monthly to add to their savings.	Counseling sessions with trained community members.
Domoney (2019)	Counseling	Mixed Methods (Surveys and Qualitative)	Package	Hospital and home; One‐on‐one	Counseling sessions separately with expectant mothers and fathers over the span of 2.5 years on topics such as safeguarding, mental illness, parent‐infant relationships, recovering from trauma, and improving parenting. Work to address their complex issues and support lasting behavior change, alongside managing the risks for each family member, and acting to address any safeguarding concerns that emerge. Therapeutic sessions are designed to assist parents to face up to past behaviors and experiences, including adverse childhood experiences, and current domestic abuse. The program integrates a range of therapeutic techniques to support behavior change and recovery from trauma, including Cognitive Behavioral Therapy, Transactional Analysis, Gestalt techniques, mindfulness and systemic practice in Motivational Interviewing. The Inner Child work is at the therapeutic core of the program.	Counseling sessions with both parents using a range of therapeutic techniques.
El‐Mohandes (2008)	Counseling	Randomized Controlled Trial	Package	Community clinic; One‐on‐one	Interventions for active and passive smoking were based on the Social Cognitive Theory and the Transtheoretical Model. The IPV intervention was adapted to individualized behavioral counseling sessions and was integrated from a brochure‐based approach using Dutton's Empowerment theory. To be considered adequate, the intervention was intended to be delivered prenatally in a minimum of four sessions, with eight sessions considered complete. Up to two postpartum booster sessions were offered, reinforcing skills and goals and adapted to specific postpartum stressors.	Behavioral counseling based on Dutton's Empowerment theory.
Florsheim (2011)	Counseling	Randomized Controlled Trial	IPV Focus	Community clinic or home; Group	The Young Parenthood Program (YPP) is a flexible, 10‐week (±2 weeks) couples‐focused prevention program designed to help pregnant women and their coparenting partners develop the interpersonal skills to establish and maintain a supportive co‐parenting alliance regardless of whether the couple lives together or remains romantically involved. The YPP is designed to be client centered and community based, in the sense that couples are met where they are most comfortable with respect to their interpersonal development. The YPP is implemented in phases and allows couples to move through the phases at varying speeds. Counselors determine how much time to spend on a program phase based on the couple's wants, needs, and goals. Phases include: (1) introduction and engagement; (2) setting personal and relationship goals; (3) develop specific relational competences and communication skills; (4) redefining and negotiating roles; (5) summing up and looking forward.	Couples‐focused counseling that is client‐centered and implemented in 5 phases.
Haberland (2020)	Counseling	Randomized Controlled Trial	IPV Focus	Hospital; One‐on‐one	The intervention included four parts: (1) provider training, (2) counseling aids for providers to use during the post‐test counseling session, (3) an IPV counselor was present to handle all IPV referrals immediately, (4) provider support group sessions. Counselors used a counseling script that elaborated the main messages on their counseling aid, which included key messages and resources regarding IPV, power, and women's health. The script guided counselors and included lines such as “We've begun giving this card to all our ANC clients so they know how to get help for themselves if they need it, or so they can help a friend or family member who might need it…” “Even if you are not in a relationship now, this is still important information for the future. I want you to think about these questions… Is your partner kind to you? Does he respect you and support you?” “Remember, you have a right to be free from violence and you deserve to be treated with respect” and “Even if you don't need any of this support now, relationships change—remember you can always come back to KNH for help.”	Access to an IPV counselor and counseling session.
Mantler (2022)^a^	Counseling	Retrospective Case Control Study	IPV Focus	Hospital; One‐on‐one	Trauma and violence‐informed cognitive behavioral therapy (TVICBT intervention). The intervention was composed of an initial assessment, two to three therapeutic relationship‐building sessions and up to eight TVICBT‐focused sessions. Sessions ranged from 60 to 90 min and were booked to enhance the convenience and accessibility of the woman.	Trauma‐informed CBT.
Jackson (2020)^a^	Counseling	Qualitative Study	IPV Focus	Hospital; One‐on‐one	Trauma and violence‐informed cognitive behavioral therapy (TVICBT intervention). The intervention was composed of an initial assessment, two to three therapeutic relationship‐building sessions and up to eight TVICBT‐focused sessions. Sessions ranged from 60 to 90 min and were booked to enhance the convenience and accessibility of the woman.	Trauma‐informed CBT.
Kan (2021)	Counseling	Randomized Controlled Trial	IPV Focus	Community center; Group	The Safe Dates program is a 10‐session, group‐based program and sessions were mostly ~50 min each. Included interactive discussions, role‐plays, and brainstorming. The risk factors and contextual issues targeted were those identified in formative research.	Group counseling involving interactive discussions and role play.
Katz (2008)	Counseling	Mixed Methods (Pre‐Test Post‐Test and Qualitative)	Package	Community clinic; One‐on‐one	10 counseling sessions during the antenatal and postpartum period, each lasting for a maximum of 45 min. Homework was assigned, for example, developing safety plans in the context of IPV.	Counseling sessions during antenatal and postpartum care.
Matseke (2013)	Counseling	Pre‐Test Post‐Test Design	IPV Focus	Community clinic; One‐on‐one	Single 20‐min counseling session including supportive care, anticipatory guidance, and guided referrals adapted from the March of Dimes protocol.	Brief counseling session and supported referrals.
Nakku (2021)	Counseling	Prospective Cohort Study	Package	Community clinic; Group	Mental Health Care Plan (MHCP) included provision of group‐based problem‐solving therapy (GPST), providing interpersonal violence support, and antidepressant medication. GPST included psychoeducation about maternal depression causes and training in problem solving skills. A minimum of 4 sessions was offered, took place monthly, and lasted for 1.5 to 2 h. IPV support was provided during at least one group session.	Group problem solving therapy targeting IPV and depression, and antidepressant medication prescription.
Subramanian (2012)	Counseling	Randomized Controlled Trial	Package	Community clinic; One‐on‐one	A single‐visit counseling intervention developed by Parker et al. for women experiencing IPV was modified to provide ongoing guidance throughout pregnancy. Eight prenatal sessions were required to deliver the complete intervention. However, a minimum of 4 sessions was deemed “adequate” on the basis of the amount of material that could be covered in 4 sessions. Women in need of other social services were provided with referrals to specific resources.	Counseling to target IPV.
Tiwari (2005)	Counseling	Randomized Controlled Trial	IPV Focus	Hospital; One‐on‐one	One time 30‐min interview. The intervention was based on an empowerment protocol and consisted of advice in the areas of safety, choice making and problem solving. A component of empathic understanding, derived from Roger's client‐centered therapy, was also added to the empowerment protocol. Empathic understanding emphasized the need to take in and accept the woman's perceptions and feelings. At the conclusion of the interview, the woman was given a brochure reinforcing the information provided.	One time counseling session focused on empowerment and empathic understanding.
Trabold (2020)	Counseling	Mixed Methods (Pre‐Test Post‐Test and Qualitative)	IPV Focus	Community clinic; One‐on‐one	Brief Negotiated Interview adapted to address IPV victimization. The first session involved a general discussion of IPV and its personal impact via a personalized information pamphlet specialized to each participant based on responses from the pretreatment measures. The positive and negative aspects of the relationship, readiness to address IPV, and/or engage in IPV services were addressed, followed by a discussion of personal strengths, resilience, and support. An action plan was offered to outline goals and a plan to achieve these goals. Two additional in‐person follow‐up sessions followed at 1 and 3 weeks from the first session. These sessions utilized motivational interviewing principles (e.g., open‐ended questions, reflections, summary statements, and affirmations) to discuss progress toward the action plan. The first two sessions took 30 to 45 min to complete, and the follow‐up sessions lasted 15 to 30 min.	Counseling sessions utilizing motivational interviewing principles.
Zlotnick (2011)	Counseling	Randomized Controlled Trial	IPV Focus	Community clinic; One‐on‐one	Four 60‐min individual sessions over a 4‐week period before delivery and followed by one 60‐min individual “booster” session within 2 weeks of delivery. Intervention was based primarily on the principles of interpersonal psychotherapy. Intervention was designed to help participants improve their significant interpersonal relationships, change their expectations about them, assist them in building or improving their social support networks, and master their role transition to motherhood.	Counseling sessions based on interpersonal psychotherapy principles.
*Education*						
Alizadeh (2021)	Education	Randomized Controlled Trial	Package	Location unspecified; Group and one‐on‐one	There were two intervention groups, Group A and Group B and both interventions featured the Sexual Health Education Program (SHEP). SHEP features educational materials including the most important dimensions of sexual health (e.g., notes about the components of the reproductive system of men and women, sexual activity and its changes in pregnancy, pre‐sex preparation and sexual intimacy, marital satisfaction, sexual restrictions during pregnancy, the right beliefs and attitudes about sexual function and correcting false beliefs and attitudes, domestic violence during pregnancy). Group A: 3 small group teachings based on SHEP, each 90 min, completed at each trimester of pregnancy. The classes were held in the form of a lecture with questions and answers. At the end of each class, the women were given a booklet to review and share with their spouses. Group B: self‐training using SHEP at each trimester.	Sexual health training program in the form of small group lectures or self‐study.
Flaathen (2022)^a^	Education	Randomized Controlled Trial	IPV Focus	Community clinic; One‐on‐one	Safe Pregnancy Study tested effectiveness of a tablet‐based, culturally adapted intervention. Intervention included a film (7 min in length) about the nature of IPV and behaviors that can increase safety.	Educational film about IPV and safety behaviors.
Walter (2021)^a^	Education	Qualitative Study	IPV Focus	Community clinic; One‐on‐one	Safe Pregnancy Study tested effectiveness of a tablet‐based, culturally adapted intervention. Intervention included a film (7 min in length) about the nature of IPV and behaviors that can increase safety.	Educational film about IPV and safety behaviors.
Kan (2015)	Education	Randomized Controlled Trial	Package	Community center; Group	Completed the Family Foundations program consisting of 4 prenatal classes to introduce skills for strengthening their relationship, coparenting, and parenting. Afterwards, there were 4 postnatal classes which revisit those themes. The group sessions involved 6–10 couples. A combination of didactic presentations, couple communication exercises, written worksheets, videotaped vignettes of other families, and group discussion was utilized.	Educational prenatal and postnatal classes centered around parenting skills.
Kiely (2010)	Education	Randomized Controlled Trial	IPV Focus	Community clinic; One‐on‐one	Individually tailored counseling sessions delivered during prenatal care specific to each of the designated psycho‐behavioral risks. The IPV intervention emphasized safety behaviors and provided information about the types of abuse, the cycle of violence, a danger assessment to assess risks, and preventive options women might consider as well as the development of a safety plan. A list of community resources with addresses and phone numbers was provided. Eight prenatal sessions was considered a complete intervention.	Education to target IPV.
Miller (2011)	Education	Randomized Controlled Trial	IPV Focus	Community clinic; One‐on‐one	Enhanced IPV screening, which focuses first on educating clients about reproductive coercion and the many forms of IPV. Reproductive health specialists (RHS) then assist the patient in identifying harm reduction behavioral strategies specific for the reason the client is visiting the clinic to reduce risk for IPV and reproductive coercion. RHS educate women regarding local IPV and sexual assault resources and facilitate utilization of these services by contacting these programs together with the client or by offering a safe space within the clinic for patients to initiate such contact. Business‐card‐size intervention cards were developed to serve both as an ongoing clinical prompt for staff and as a resource for patients.	Education paired with referrals to community organizations.
Rishal (2020)	Education	Pre‐Test Post‐Test Design	IPV Focus	Hospital; One‐on‐one	Teaching women 15 safety measures using a flip chart of text and illustrations. Teaching sessions lasted for a maximum of 30 min.	Education about safety measures via text and illustrations.
Taft (2015)	Education	Randomized Controlled Trial^c^	IPV Focus	Community center; One‐on‐one	The Maternal and Child Health Care for Vulnerable Mothers (MOVE) intervention is an enhanced screening and care model. It featured a self‐completion maternal health and wellbeing checklist to screen for DV and a clinical pathway/guidelines for screening nurses to follow. Nurse mentors were present to assist with nurse safety, support colleagues with difficult consultations, and enhance liaison with DV services. Family violence liaison workers were present to facilitate referrals to family violence services.	Provider training program to enhance screening for DV and appropriate referrals to family violence services.
Taghizadeh (2018)	Education	Randomized Controlled Trial	IPV Focus	Community clinic; Group	Four problem‐solving skills training sessions were scheduled (one session per week, lasting 90 min). Training was held in groups and the techniques used included class activity, role play, and home assignments. Each new session began with an overview of the materials taught in the previous session and an assessment of the home assignments and ended with a Q&A.	Problem‐solving skills training sessions.
Turan (2013)	Education	Mixed Methods (Surveys, Focus Groups, and Interviews)	IPV Focus	Community clinic; One‐on‐one	The intervention included three parts: building the skills of local community partners to respond to GBV, training all clinic staff through a 40‐h training program, and finally offering supported referrals to services that already exist in the province. Supported referrals made included referrals to community referral persons, police in the nearest town, local government administrators (to help with needs for shelter and food), nongovernmental organizations working on women's rights (for counseling), a probono lawyer (for those who wanted to pursue legal action), and village elders (for help with communication with the husband and family). Supported referrals are distinct from ordinary referrals because community volunteers offer concrete assistance for reaching referral services in the community or the nearest town, including provision of transport costs, personally escorting women to services, telephoning ahead and offering emotional support.	Training local community services and healthcare workers; referrals to various support resources in the region.
Van Parys (2017)	Education	Randomized Controlled Trial	IPV Focus	Hospital; One‐on‐one	Intervention consisted of three parts: a questionnaire, a referral card, and two interviews. The questionnaire was used to invite women to participate in the intervention. Women were handed an envelope at their 6‐week postpartum consultation. The envelope contained an information letter and a bank card‐sized referral card containing the contact details of services providing assistance for IPV on one side and tips to increase safety behavior. The participants were interviewed 10 to 12 months and 16 to 18 months after receipt of the envelope.	Referral card with community resources and tips to increase safety behavior.
*Counseling and Education*						
Akor (2019)	Counseling; Education	Randomized Controlled Trial	IPV Focus	Hospital; One‐on‐one	Three counseling sessions provided biweekly during routine antenatal visits. Counseling was done using the SOS‐DoC framework (S—offer support and assess safety; O—discuss options; S—validate patient's strengths; Do—document observations, assessment, and plans; C—offer continuity). The framework was individualized for each victim and depending on what was reported as the likely cause of the IPV suggestions were made to resolve such problems.	Counseling sessions featuring safety planning.
Arora (2019)	Counseling; Education	Pre‐Test Post‐Test Design	IPV Focus	Hospital; One‐on‐one	Minimum of 2 counseling sessions provided during antenatal care visits, lasting 30–45 min. Sessions consisted of empathetic listening, provision of emotional support, safety planning, assisting with filing police complaints, and helping with referrals to other support services. Safety assessment and planning was provided.	Counseling sessions during antenatal care visits paired with safety planning and supported referrals.
Cripe (2010)	Counseling; Education	Randomized Controlled Trial	IPV Focus	Hospital; One‐on‐one	Referral card, supportive counseling and education, and advice in the areas of safety by a trained social worker for 30 min. Interviewers helped women understand the cycle of violence and reviewed components of the safety plan. Participants were given a brochure with a 13‐item safety plan to reinforce the safety behaviors and were provided with a list of community resources. Interviewers offered to assist women with telephone calls to social service agencies or women's groups who could act as advocates for abused women. Each woman received supportive care, validation of feelings, empathetic listening, and information on what to expect when seeking help from legal resources, shelters, law enforcement, or counseling services.	Counseling sessions paired with education and supported referrals to community organizations.
Curry (2006)	Counseling; Education	Randomized Controlled Trial	IPV Focus	Community clinic; One‐on‐one	All participants were offered the video Faces of Abuse, and 24/7 access to a Connections Nurse Case Manager (NCM). Women were offered a bright refrigerator magnet with the Connections logo, the 24/7 phone number, and a letter explaining the NCM services. At least one phone call was made to all women at low risk, with most contacted to remind them of the services. All women at high risk were called until contact was made with the NCM. All case‐managed women received an initial, comprehensive assessment to develop an individualized care plan based on needs they identified. Nurses engaged in interventions coded into six categories: support (e.g., provide emotional support), assess (e.g., food, shelter), educate (e.g., stress management skills), monitor (e.g., current medical treatment plan), coordinate (e.g., locate resources for client), and coach (e.g., review personal safety plan).	Nurse case management providing support, assessment, education, monitoring, coordination, and coaching.
Dinmohammadi (2021)	Counseling; Education	Randomized Controlled Trial	IPV Focus	Community center; One‐on‐one	90‐min Individual counseling sessions weekly for 6 weeks. Sessions teach solution‐focused approaches, define problems, educate about quality of life, framing events, and recognizing one's own destructive behavior patterns.	Counseling sessions including education.
Khalili (2020)	Counseling; Education	Randomized Controlled Trial	IPV Focus	Community clinic; One‐on‐one	Four sessions of supportive‐educational intervention, based on content such as discussing violence cycle, practicing emotional disclosure and emotional release and training problem‐solving and conflict resolution skills. The session occurred twice a week lasted 60–90 min. At the beginning of each session, the objective was described. Each session was initiated by reviewing the previous meeting, continued with presenting of the specified content, answering questions, and clarifying ambiguities and finally, session ended with setting up the next session.	Counseling and education intervention.
Kramer (2012)	Counseling; Education	Pre‐Test Post‐Test Design	IPV Focus	Hospital and community clinic and home; One‐on‐one	Safe Mom, Safe Baby is a nurse‐led interdisciplinary clinical program that provides comprehensive, fully integrated services to pregnant and recently delivered clients experiencing IPV. It is an interdisciplinary program that combines registered nurse case management, a community‐based domestic violence agency, and perinatal care. Services include crisis intervention, safety planning, emotional support, mental health and substance use support, advocacy, legal and police orders of protection and basic needs (formula, transport, clothes, etc.).	Nurse case management, medical care, and support from a community domestic violence agency.
Laughon (2011)	Counseling; Education	Pre‐Test Post‐Test Design	IPV Focus	Community clinic; One‐on‐one	Brief Negotiated Interview adapted for IPV victimization, based on March of Dimes. Counseling sessions lasted 10 min and consisted of four components: (a) IPV information, (b) Danger Assessment, (c) Safety Planning & Options, and (d) Resources.	Counseling sessions focused on education and safety planning.
McFarlane (2000)	Counseling; Education	Randomized Controlled Trial	IPV Focus	Community clinic; One‐on‐one	Three intervention groups: (1) Brief – received resource card with phone numbers of local agencies to assist in domestic abuse; (2) Counseling – unlimited access to counseling with expert in domestic abuse (3) Outreach – unlimited access to professional counselor AND mentor mother. The role of the mentor was to offer support, education, referral, and assistance in accessing community resources through personal visits and telephone contacts with the abused women.	Education, counseling, or both.
Miller (2016)	Counseling; Education	Randomized Controlled Trial^c^	IPV Focus	Community clinic; One‐on‐one	Clinicians and staff at intervention clinics received a half‐day ARCHES training from IPV victim service advocates. Discussion of IPV/reproductive coercion (RC) was encouraged for all encounters via provision of a palm‐sized brochure to every client about the health impact of IPV/RC, harm reduction (e.g., intrauterine and emergency contraception, safety planning to reduce risk for IPV), and IPV resources. The intervention typically took less than a minute. When IPV or RC was disclosed, counseling to reduce risk of partner interference with contraception and increase safety required additional time, including making a referral to an advocate if the client was interested. All participants were offered a women's health resource sheet.	Counseling sessions paired with education and referrals to community organizations.
Mutisya (2018)	Counseling; Education	Quasi‐Experimental Study	IPV Focus	Hospital; One‐on‐one	The psychosocial intervention entailed provision of unbiased information on the meaning and magnitude of GBV, the GBV cycle and the adverse effects of violence during pregnancy. Research assistants actively interviewed, listened empathetically, validated feelings, encouraged the participants, and pointed out the woman was not to blame for the violence meted on her. The intervention included a safety assessment and facilitation to identify measures that participants could take to enhance their safety based. Participants received resource cards listing local persons/organizations from where they could seek further assistance for GBV in addition to information on the availability of coordinated referrals for specialized counseling services. The research assistants aimed to hold at least three 30–35 min psychosocial support sessions with each participant before conducting the last intervention interview. The first session took place within 2–3 weeks after the baseline interview and the remaining sessions spread over a 4–5 month period. The sessions coincided with ANC appointment dates whenever possible and special appointments were negotiated where the former was not possible and in case of missed antenatal care visits. Transportation cost was reimbursed, and participants were given a snack whenever they had a special appointment.	Counseling sessions that included safety planning and education about available resources.
Rowe (2014)	Counseling; Education	Quasi‐Experimental Study	Package	Home; One‐on‐one	The Survivor Moms' Companion is a primary care, fully manualized, ten module, self‐study and structured‐listening program designed to provide psychoeducation. It also provides case‐finding for the estimated 10% to 15% of people who will also benefit from referral for treatment with medication or psychotherapy. The self‐study program is structured with workbook modules that the woman works on to prepare for a 30‐min structured telephone consultation with a tutor.	Self‐study and structured‐listening program to provide psychoeducation.
Sapkota (2022)	Counseling; Education	Randomized Controlled Trial	IPV Focus	Hospital; One‐on‐one	Intervention based on the Problem Management Plus and the Safe and Sound interventions. Face to face counseling session guided by motivational interviewing techniques was completed, where women were encouraged to express their feelings and concerns by asking questions and a counselor assisted them to develop plans. Women were also provided with an information booklet with info about IPV, mental health impacts, strategies to reduce stressors, safety tips and local support organizations. The participants were provided with the contact details of the counselor so they could contact her if needed during the study period.	Counseling and education in the form of an information booklet; allowed to contact counselor during study period.
Sapkota (2020)	Counseling; Education	Qualitative Study	IPV Focus	Hospital; One‐on‐one	Intervention based on the Problem Management Plus and the Safe and Sound interventions. Face to face counseling session guided by motivational interviewing techniques was completed, where women were encouraged to express their feelings and concerns by asking questions and a counselor assisted them to develop plans. Women were also provided with an information booklet with info about IPV, mental health impacts, strategies to reduce stressors, safety tips and local support organizations. The participants were provided with the contact details of the counselor so they could contact her if needed during the study period.	Counseling and education in the form of an information booklet; allowed to contact counselor during study period.
Zlotnick (2019)	Counseling; Education	Randomized Controlled Trial	IPV Focus	Hospital; One‐on‐one	Computerized intervention (30–40 min) on a tablet, called SURE. SURE is theory‐driven and consistent with motivational interviewing principles. SURE presents information and education regarding types of IPV, associated risks for the woman, fetus, and offspring; potential health problems associated with relationship abuse; and risks of untreated mental health problems. SURE emphasizes the bidirectional relationship between mental health and IPV and the narrator assessed the participant's readiness to utilize resources (e.g., remain in mental health treatment, use IPV hotlines, talk to health care provider/support person about IPV and IPV resources). Participants had the option to create a personalized safety plan that offered tailored advice for decision‐making that maximizes their safety and had the option of selecting from a menu of potential personal change goals to learn more about specific topics, such as building support for IPV, building self‐esteem, safety planning for IPV, breaking up with an abusive partner, how to talk to mental health care provider about seeking IPV resources and/or managing anger towards abuser. These topics were presented as a series of empowerment videos that depicted women presenting on a topic, how they managed (or skills they used for) a specific IPV‐related issue, and related positive outcome. Women were provided with optional printouts of related materials from the intervention as a resource as well as empowerment messages reinforcing the video content.	Computerized intervention providing education about IPV harms and automated counseling.
Rastegar (2021)	Counseling; Education	Randomized Controlled Trial	IPV Focus	Community clinic; Group and one‐on‐one	Five sessions of public health educations from a clinical psychologist for 5 weeks at the health care clinic. Education related to IPV identification, booklet containing information about IPV and conflict management techniques, group discussion, and watched a movie about DV. The women received consultation to improve their communication by developing interpersonal skills. Contact details of the researchers were provided and the women were encouraged to contact and seek advice to manage their family issues as needed. The women were also urged to actively seek a supportive environment among friends and family members, particularly with their mothers. They were also asked to develop their relationship with the support group at the clinic and the research team during the study period.	Education sessions using various modalities and including group discussion and support from a clinical psychologist.
*Home Visitation*						
Fergusson (2005)^a^	Home Visitation	Randomized Controlled Trial	Package	Home; One‐on‐one	The Early Start program provides intensive home visiting to families facing multiple challenges. Home visiting is provided by trained family support workers with qualifications in nursing, teaching, or allied disciplines. It uses a social learning model, whose critical elements include (1) assessment of family needs, issues, challenges, strengths, and resources; (2) development of a positive partnership between the family support worker and client; (3) collaborative problem solving to devise solutions to family challenges; (4) the provision of support, mentoring, and advice to assist client families to mobilize their strengths and resources; and (5) involvement with the family throughout the child's preschool years.	Home visitation based on problem solving with the family, while providing mentorship.
Fergusson (2013)^a^	Home Visitation	Randomized Controlled Trial	Package	Home; One‐on‐one	The Early Start program provides intensive home visiting to families facing multiple challenges. Home visiting is provided by trained family support workers with qualifications in nursing, teaching, or allied disciplines. It uses a social learning model, whose critical elements include (1) assessment of family needs, issues, challenges, strengths, and resources; (2) development of a positive partnership between the family support worker and client; (3) collaborative problem solving to devise solutions to family challenges; (4) the provision of support, mentoring, and advice to assist client families to mobilize their strengths and resources; and (5) involvement with the family throughout the child's preschool years.	Home visitation based on problem solving with the family, while providing mentorship.
Duggan (2004)	Home Visitation	Randomized Controlled Trial	Package	Home; One‐on‐one	Healthy Start Program (HSP) model has two components: (1) population‐based screening and assessment of families of newborns to identify those at‐risk of child abuse and neglect, and (2) long‐term, intensive home visiting of at‐risk families by trained paraprofessionals in the child's first 3–5 years of life. Visitors are to model problem‐solving skills and help families access to community resources.	Home visitation assisting with community service access.
Olds (2004)^a^	Home Visitation	Randomized Controlled Trial	Package	Home; One‐on‐one	Women assigned to the paraprofessional group were provided developmental screening and referral services for their child plus paraprofessional home visitation during pregnancy and infancy (the first 2 years of the child's life). Women in the professional (nurse) group were provided screening and referral plus nurse home visitation during pregnancy and infancy.	Paraprofessional or professional (nurse) home visitation paired with developmental screening and referrals for children's health. Nurse‐Family Partnership and paraprofessional programs.
Olds (2007)^a^	Home Visitation	Randomized Controlled Trial	Package	Home; One‐on‐one	Women assigned to treatment 3 were provided free round‐trip taxicab transportation for scheduled prenatal care appointments plus intensive nurse home‐visiting services during pregnancy, 1 post‐partum visit in the hospital before discharge, and 1 post‐partum visit in the home. Women assigned to treatment 4 received the same services as treatment 3, however also continued to be visited by nurses until the child's second birthday.	Professional (nurse) home visitation paired with free transportation to prenatal care appointments and additional post‐partum appointments. Nurse‐Family Partnership.
Olds (2010)^a^	Home Visitation	Randomized Controlled Trial	Package	Home; One‐on‐one	Women were provided free transportation for scheduled prenatal care plus developmental screening and referral services for their child at age 6, 12, and 24 months. They were also provided professional (nurse) home visitation until their child's second birthday.	Professional (nurse) home visitation paired with free transportation to prenatal care appointments. Nurse‐Family Partnership.
Taft (2011)	Home Visitation; Counseling; Education	Randomized Controlled Trial^c^	IPV Focus	Home and community center; One‐on‐one	In MOSAIC (MOtherS' Advocates In the Community), women received up to 12 months of support from trained and supported non‐professional mentor mothers. Research staff gave all women a resource card for new mothers, which included contacts for family violence services. Support from mentor mothers included: providing non‐judgemental listening, support and friendship; maintaining contact (weekly on average) and support through phone calls, home visiting and outings; assistance in developing safety strategies appropriate to women's circumstances; developing a trusting relationship and modeling a sense of hope; providing information and support with parenting; and providing information about, and assisted referral to community services (especially family violence services) and resources for women and their children.	MOSAIC provides women with support from mentor mothers. Includes providing support and non‐judgmental listening, home visiting and outings, education, and assisted referral to community resources.
Ammerman (2022)	Home Visitation; Education	Randomized Controlled Trial	Package	Home; Group and one‐on‐one	Family Foundations + Home Visiting (FFHV) is an 11‐visit model and sessions consisted of discussion topics, individual and couples' exercises, and practice. Sessions reflected four overarching themes: self‐regulation, child needs, coparenting, and parental adjustment. Video vignettes were shown to demonstrate specific parenting approaches and ways of negotiating parenting cooperation. Incorporated two separate one‐on‐one sessions between fathers and the male home visitor to focus on fathers' needs and encourage engagement in the program.	Home visitation featuring discussions with interventionists, individual and couples' exercises, and practice.
Barlow (2007)^a^	Home Visitation; Education	Randomized Controlled Trial	Package	Home; One‐on‐one	18 months (6 months antenatally to 12 months postnatally) of weekly visits from a health visitor trained in understanding the processes of helping, skills of relating to parents effectively, and methods of promoting parent‐infant interaction using the Family Partnership Model.	Home visitation providing support, and education using the Family Partnership Model.
McIntosh (2009)^a^	Home Visitation; Education	Randomized Controlled Trial	Package	Home; One‐on‐one	18 months (6 months antenatally to 12 months postnatally) of weekly visits from a health visitor trained in understanding the processes of helping, skills of relating to parents effectively, and methods of promoting parent‐infant interaction using the Family Partnership Model.	Home visitation providing support, and education using the Family Partnership Model.
Feder (2018)	Home Visitation; Education	Randomized Controlled Trial	IPV Focus	Home; One‐on‐one	Intervention contained three main components: nurse training and screening assessment of IPV, secondary prevention component, and primary prevention component. Secondary prevention component was a brochure that included a discussion of power and control; administration of the Danger Assessment Scale; and the development of a client‐driven safety plan, along with information on national and local resources for IPV. Primary prevention component was adapted from the Within My Reach curriculum and included 5 units focusing on understanding, building, and maintaining healthy relationships, and each unit included skills‐based activities that created opportunities to learn and practice conflict management, communication, and decision‐making skills. Women had the opportunity to observe and practice these skills through role‐playing with their nurse.	IPV prevention educational intervention in a nurse home visitation program.
Jack (2019)	Home Visitation; Education	Randomized Controlled Trial	IPV Focus	Home; One‐on‐one	Nurses received intensive IPV education and delivered an IPV intervention that included a clinical pathway to guide decision making that included elements of assessment, diagnosis, care planning, and intervention tailored to the participant's needs. This tailored intervention consisted of (1) universal assessment of safety or case‐finding assessment approaches to identify IPV exposure and (2) empathic response to IPV disclosure, followed by (3) risk assessment (administration of the Danger Assessment instrument) plus an adapted brief empowerment intervention including immediate discussion of safety options and (4) assessment of mental health, substance use, and stage of readiness to address safety to plan a nursing response tailored to a woman's needs that focuses on (5) safety, awareness of IPV health effects, self‐efficacy, and system navigation. System navigation involves identifying, referring, and actively facilitating participant access to external domestic violence, legal, housing, or other health or social care services.	Home visitation featuring educational resources and referrals to community resources. Nurse‐Family Partnership.
Jacobs (2016)	Home Visitation; Education	Randomized Controlled Trial	Package	Home; One‐on‐one	Healthy Families Massachusetts provides home visiting services that include goal setting, curriculum‐based activities and family support tailored to individual families, routine developmental and health screenings, and linkages to medical and other services as needed. Parents can enroll from pregnancy until the child's first birthday, with services available through the child's third birthday. Biweekly visits are intended for prenatal participants, and weekly visits are expected for at least 6 months after the birth of the baby or following enrollment for participants who enter the program after childbirth.	Home visitation providing educational activities, health screenings, and linkages to medical and community services.
Mejdoubi (2013)	Home Visitation; Education	Randomized Controlled Trial	IPV Focus	Home; One‐on‐one	Offered 10 nurse home visits during pregnancy by experienced VoorZorg nurses. Six domains (health status of the mother, child's health and safety, personal development of the mother, the mother as a role model, relation of the mother with her partner, family and friends, and use of institutions) were addressed during each visit. The VoorZorg nurses attempted to mitigate risk factors for IPV by reducing stress, by trying to make women financially independent, or by providing housing assistance. Nurses helped women (and their partners) during home visits to be aware of IPV, to identify abusive relationships by use of the Power and Control Wheel and to make them aware of the consequences of abuse for the child. VoorZorg nurses supported women and their partners with strategies for emotional regulation and communication. The nurses also helped both partners to make safer decisions for the sake of themselves and their child, such as preventing arguments from escalating to a physical fight by teaching them how to address these situations, and by teaching them how to negotiate and to listen to each other.	Home visitation featuring educational discussions. Nurse‐Family Partnership.
Sharps (2016)^a^	Home Visitation; Education	Randomized Controlled Trial	IPV Focus	Home; One‐on‐one	The Domestic Violence Enhanced Home Visitation Program (DOVE) is a structured brochure based IPV empowerment intervention based on the March of Dimes Protocol and was developed to be integrated within home visiting programs. The DOVE intervention was delivered six times within regularly scheduled home visits, three sessions occurring during pregnancy and three during the postpartum period, each session lasting 15–25 min. Each intervention session included the home visitor reviewing the DOVE brochure that contained information addressing the cycle of violence, the Danger Assessment that assessed risk factors of homicide, choices available to the woman, safety planning information tailored to the context and level of danger, and IPV resources specific to each community, as well as national hotline information. Home visitors tailored the intervention to her expressed needs and level of danger at each visit. In the second group, the materials are delivered using mHealth (i.e., a computer tablet).	Home visitation featuring educational resources. Includes Nurse‐Family Partnership and paraprofessional programs.
Bacchus (2016) (Infusing technology)^a^	Home Visitation; Education	Qualitative Study	IPV Focus	Home; One‐on‐one	The Domestic Violence Enhanced Home Visitation Program (DOVE) is a structured brochure based IPV empowerment intervention based on the March of Dimes Protocol and was developed to be integrated within home visiting programs. The DOVE intervention was delivered six times within regularly scheduled home visits, three sessions occurring during pregnancy and three during the postpartum period, each session lasting 15–25 min. Each intervention session included the home visitor reviewing the DOVE brochure that contained information addressing the cycle of violence, the Danger Assessment that assessed risk factors of homicide, choices available to the woman, safety planning information tailored to the context and level of danger, and IPV resources specific to each community, as well as national hotline information. Home visitors tailored the intervention to her expressed needs and level of danger at each visit. In the second group, the materials are delivered using mHealth (i.e., a computer tablet).^a^ This study explores the perspectives of the second group (mHealth).	Home visitation featuring educational resources. Includes Nurse‐Family Partnership and paraprofessional programs.
Bacchus (2016) (Opening the door)^a^	Home Visitation; Education	Qualitative Study	IPV Focus	Home; One‐on‐one	The Domestic Violence Enhanced Home Visitation Program (DOVE) is a structured brochure based IPV empowerment intervention based on the March of Dimes Protocol and was developed to be integrated within home visiting programs. The DOVE intervention was delivered six times within regularly scheduled home visits, three sessions occurring during pregnancy and three during the postpartum period, each session lasting 15–25 min. Each intervention session included the home visitor reviewing the DOVE brochure that contained information addressing the cycle of violence, the Danger Assessment that assessed risk factors of homicide, choices available to the woman, safety planning information tailored to the context and level of danger, and IPV resources specific to each community, as well as national hotline information. Home visitors tailored the intervention to her expressed needs and level of danger at each visit. In the second group, the materials are delivered using mHealth (i.e., a computer tablet).^a^ This study explores the perspectives of the first group (non‐mHealth).	Home visitation featuring educational resources. Includes Nurse‐Family Partnership and paraprofessional programs.
McConnell (2020)	Home Visitation; Counseling; Education	Mixed Methods (Surveys and Interviews)	Package	Home; Group	Steps to Safety (S2S) is a home‐based intervention designed for both heterosexual and same‐sex couples. First, patients are screened, discuss safety planning, and undergo an assessment. S2S then begins with goal setting, and the introduction of core activities focused on emotional regulation. Further sessions to support emotional regulation are delivered in parallel with Video Interaction Guidance sessions. The S2S manual is divided into five modules that comprise a total of 26 sessions. Program designed to be delivered over the course of 6 months. The program ends with parenting and closing sessions.	Home visitation featuring educational activities and counseling.

a Paired studies were conducted to evaluate the same intervention.

b RCT with pre‐ and post‐test comparison.

c Cluster RCT.

##### Intervention type

Of the 57 unique interventions, most (*n* = 39) were designed specifically to target IPV and related outcomes. For example, Van Parys et al. (2017) designed a referral card‐based intervention and measured its effect on IPV revictimization, psychosocial health, and help‐seeking and safety behaviors. However, many studies described interventions designed to target many psychosocial risks, one of which was IPV and related measures (e.g., revictimization, adverse maternal health outcomes) (*n* = 18). For example, El‐Mohandes et al. (2008) employed an integrated intervention to target multiple behavioral and psychosocial post‐partum risks including smoking, environmental tobacco smoke exposure, depression, and IPV.

Interventions were classified according to type, including cash transfer, home visitation, counseling, and education. Of the 57 interventions, most (*n* = 32) employed a single intervention type of either cash transfer (*n* = 1) (Peterman et al., 2022), home visitation (*n* = 3) (Duggan et al., 2004; Fergusson et al., 2005; Olds et al., 2004), counseling (*n* = 18) (Abrahams et al., 2022; Babaheidarian et al., 2021; Bacchus et al., 2010; Bahadir‐Yilmaz & Öz, 2018; Belaid et al., 2021; Domoney et al., 2019; El‐Mohandes et al., 2008; Florsheim et al., 2011; Haberland et al., 2020; Kan et al., 2021; Katz et al., 2008; Mantler et al., 2022; Matseke & Peltzer, 2013; Nakku et al., 2021; Subramanian et al., 2012; Tiwari et al., 2005; Trabold et al., 2020; Zlotnick et al., 2011), or education (*n* = 10) (Alizadeh et al., 2021; Flaathen et al., 2022; Kan & Feinberg, 2015; Kiely et al., 2010; Miller et al., 2011; Rishal et al., 2020; Taft et al., 2015; Taghizadeh et al., 2018; Turan et al., 2013; Van Parys et al., 2017).

A total of 25 studies employed a multi‐type intervention. Fifteen studies evaluated an intervention combining counseling and education (Akor et al., 2019; Arora et al., 2019; Cripe et al., 2010; Curry et al., 2006; Dinmohammadi et al., 2021; Khalili et al., 2020; Kramer et al., 2012; Laughon et al., 2011; McFarlane et al., 2000; Miller et al., 2016; Mutisya et al., 2018; Rastegar et al., 2021; Rowe et al., 2014; Sapkota et al., 2022; Zlotnick et al., 2019). Seven interventions combined home visitation with education (Ammerman et al., 2022; Barlow et al., 2007; Feder et al., 2018; Jack et al., 2019; Jacobs et al., 2016; Mejdoubi et al., 2013; Sharps et al., 2016). One combined cash transfer with education (Briaux et al., 2020), and two combined home visitation, counseling, and education (McConnell et al., 2020; Taft et al., 2011).

##### Intervention setting and modality

Interventions were classified according to setting, and were conducted in one of four settings, or a combination thereof: (i) in‐hospital (includes hospital and outpatient clinics located in a hospital); (ii) community clinic (medical clinic located in the community, i.e., not directly affiliated with a hospital); (iii) community center (community facility that does not provide medical care); (iv) home.

The majority of included interventions were conducted in community clinics (*n* = 20) (Abrahams et al., 2022; Bacchus et al., 2010; Curry et al., 2006; El‐Mohandes et al., 2008; Flaathen et al., 2022; Katz et al., 2008; Khalili et al., 2020; Kiely et al., 2010; Laughon et al., 2011; Matseke & Peltzer, 2013; McFarlane et al., 2000; Miller et al., 2011, 2016; Nakku et al., 2021; Rastegar et al., 2021; Subramanian et al., 2012; Taghizadeh et al., 2018; Trabold et al., 2020; Turan et al., 2013; Zlotnick et al., 2011). Meanwhile, 12 interventions were implemented in‐hospital (Akor et al., 2019; Arora et al., 2019; Babaheidarian et al., 2021; Cripe et al., 2010; Haberland et al., 2020; Mantler et al., 2022; Mutisya et al., 2018; Rishal et al., 2020; Sapkota et al., 2022; Tiwari et al., 2005; Van Parys et al., 2017; Zlotnick et al., 2019). A total of 13 interventions were conducted solely in survivors' homes (Ammerman et al., 2022; Barlow et al., 2007; Duggan et al., 2004; Feder et al., 2018; Fergusson et al., 2005; Jack et al., 2019; Jacobs et al., 2016; McConnell et al., 2020; Mejdoubi et al., 2013; Olds et al., 2004; Peterman et al., 2022; Rowe et al., 2014; Sharps et al., 2016). Five studies were conducted solely at a community center (Belaid et al., 2021; Dinmohammadi et al., 2021; Kan & Feinberg, 2015; Kan et al., 2021; Taft et al., 2015).

Six studies were conducted in multiple settings. There were two with the combination of at home and at a community center (Briaux et al., 2020; Taft et al., 2011), one in‐hospital and at a community clinic (Bahadir‐Yilmaz & Öz, 2018), one in‐hospital and at home (Domoney et al., 2019), one at a community clinic or at home depending on the survivor's circumstance (Florsheim et al., 2011), and one in‐hospital and at a community clinic and at home (Kramer et al., 2012). One intervention did not adequately describe their intervention setting for purposes of our classification and was conducted in comprehensive health centers (Alizadeh et al., 2021). All studies were conducted solely in‐person, however two interventions included both an in‐person component and telephone calls with providers (McFarlane et al., 2000; Rowe et al., 2014). There were three studies that featured interventions that were delivered via a tablet (Flaathen et al., 2022; Sharps et al., 2016; Zlotnick et al., 2019).

Of the 57 interventions, 45 featured an intervention that was conducted one‐on‐one, where the provider interacted solely with the survivor. Meanwhile, eight were conducted in a group format. Of these, four featured group sessions with multiple survivors (Belaid et al., 2021; Kan et al., 2021; Nakku et al., 2021; Taghizadeh et al., 2018). Two interventions featured couples counseling with one couple at a time (Florsheim et al., 2011; McConnell et al., 2020) and one featured couples counseling in a group of couples (Kan & Feinberg, 2015). Finally, one study featured counseling sessions where the survivor was asked to choose a member of their family to attend the sessions (e.g., a spouse, mother, or mother‐in‐law) (Babaheidarian et al., 2021).

Four studies featured a combination of group and one‐on‐one formats. The intervention employed in Briaux et al., 2020 consisted of home visits and group meetings with trained providers discussing essential family practices. The intervention featured in Alizadeh et al. (2021) consisted of two intervention groups, one of which employed small group teachings with multiple survivors, and another which consisted of survivor self‐studying. One intervention consisted of both individual exercises for the survivor as well as couples' exercises (Ammerman et al., 2022). The intervention conducted by Rastegar et al., 2021 featured group education sessions with multiple survivors as well as one‐on‐one time between the survivors and researchers.

Interventions were delivered by research assistants, nurses, trained home visitors, counselors, social workers, midwives, or a combination thereof. However, reporting on who administered the intervention was inconsistent across studies. Frequently, the intervention was administered by a research assistant who was also a professional or paraprofessional. Additionally, the person who delivered the intervention was often not specified.

#### 5.4.5 Outcome characteristics

**Figure 2:**
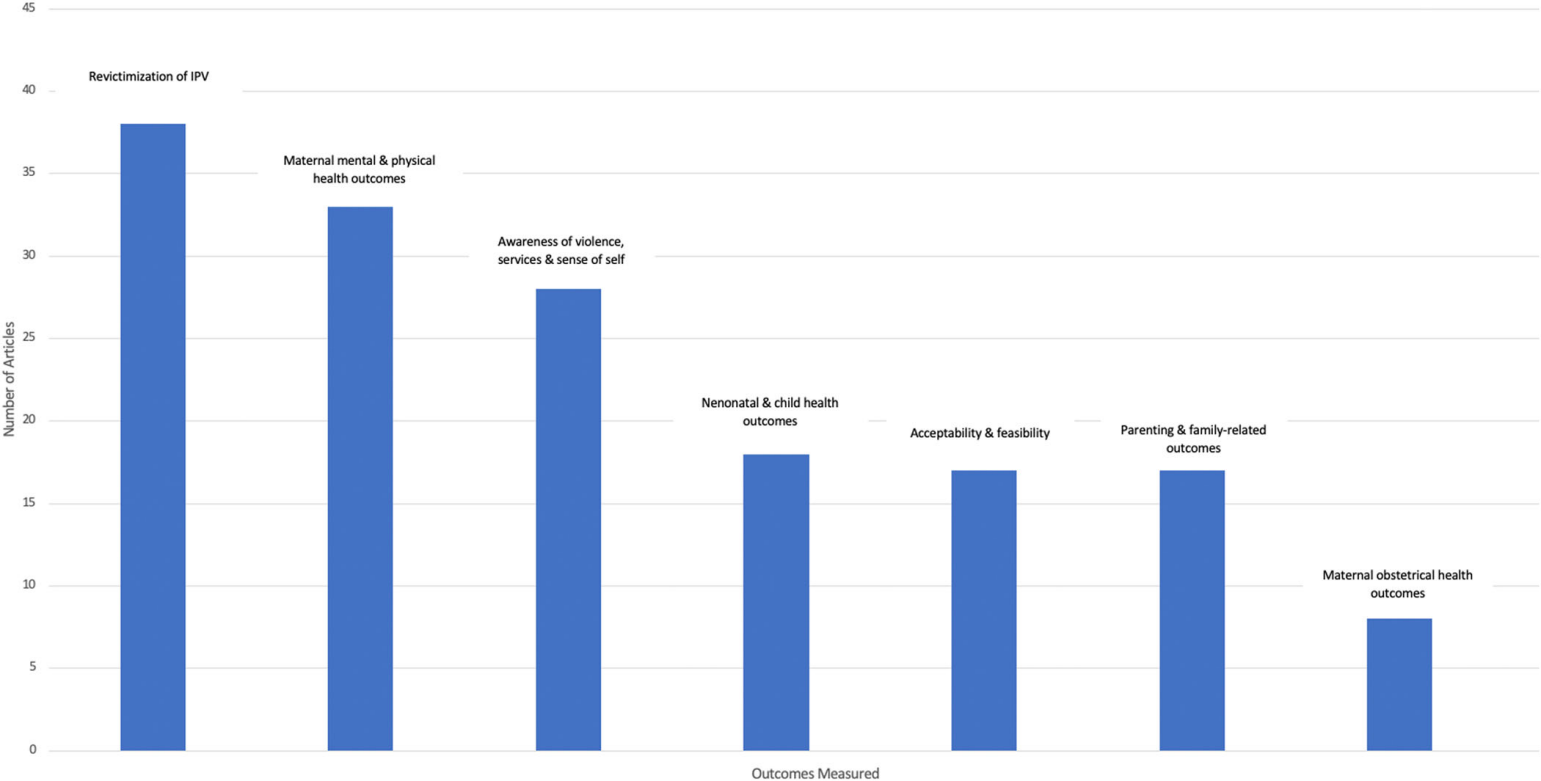
Outcomes measured in included studies.

Detailed information on measured outcomes in each included study can be found in Figure 2 and Table 3.

**Table 3:** Measured outcomes in included studies.

Measured outcome	Included studies
Revictimization of IPV	
Revictimization of All Forms of IPV	Babaheidarian (2021), El‐Mohandes (2008), Feder (2018), Fergusson (2005), Flaathen (2022), Jacobs (2016), Kan (2021), Laughon (2011), Mantler (2022), Matseke (2013), Mejdoubi (2013), Miller (2011), Mutisya (2018), Rastegar (2021), Rishal (2020), Sharps (2016), Taft (2011), Taghizadeh (2018), Tiwari (2005), Trabold (2020), Van Parys (2017), Zlotnick (2011), Zlotnick (2019)
Revictimization of Physical Violence	Ammerman (2022), Babaheidarian (2021), Bacchus (2016), Belaid (2021), Briaux (2020), Cripe (2010), Duggan (2004), El‐Mohandes (2008), Feder (2018), Flaathen (2022), Florsheim (2011), Jacobs (2016), Kan (2021), Kiely (2010), Laughon (2011), Mantler (2022), Matseke (2013), McFarlane (2000), Mejdoubi (2013), Miller (2011), Miller (2016), Mutisya (2018), Olds (2004), Olds (2007), Olds (2010), Peterman (2022), Rastegar (2021), Rishal (2020), Sharps (2016), Taft (2011), Taghizadeh (2018), Tiwari (2005), Trabold (2020), Van Parys (2017), Zlotnick (2011), Zlotnick (2019)
Revictimization of Psychological Violence	Ammerman (2022), Babaheidarian (2021), Briaux (2020), Cripe (2010), Duggan (2004), El‐Mohandes (2008), Feder (2018), Flaathen (2022), Jacobs (2016), Kan (2021), Laughon (2011), Mantler (2022), Matseke (2013), McFarlane (2000), Mejdoubi (2013), Miller (2011), Mutisya (2018), Peterman (2022), Rastegar (2021), Rishal (2020), Sharps (2016), Taft (2011), Taghizadeh (2018), Tiwari (2005), Trabold (2020), Van Parys (2017), Zlotnick (2011), Zlotnick (2019)
Revictimization of Sexual Violence	Alizadeh (2021), Babaheidarian (2021), Cripe (2010), El‐Mohandes (2008), Feder (2018), Flaathen (2022), Jacobs (2016), Kan (2021), Kiely (2010), Laughon (2011), Mantler (2022), Matseke (2013), Mejdoubi (2013), Miller (2011), Miller (2016), Mutisya (2018), Rastegar (2021), Rishal (2020), Sharps (2016), Taft (2011), Taghizadeh (2018), Tiwari (2005), Trabold (2020), Van Parys (2017), Zlotnick (2011), Zlotnick (2019)
Reciprocal Perpetration of Violence	Feder (2018), Jacobs (2016), Mejdoubi (2013)
Maternal Mental & Physical Health	
Mental Health, Unspecified	Arora (2019), Barlow (2007), Dinmohammadi (2021), Jack (2019), Olds (2004), Sapkota (2020), Tiwari (2005)
Depression	Ammerman (2022), Duggan (2004), El‐Mohandes (2008), Jack (2019), Mantler (2022), Mutisya (2018), Nakku (2021), Olds (2007), Poleschuk (2019), Rowe (2014), Sapkota (2022), Sharps (2016), Taft (2011), Trabold (2020), Zlotnick (2011)
PTSD	Ammerman (2022), Jack (2019), Mantler (2022), Trabold (2020), Zlotnick (2011)
Substance Use	Curry (2006), Duggan (2004), El‐Mohandes (2008), Jack (2019), Jacobs (2016), Mantler (2022), Olds (2004), Olds (2007), Olds (2010), Sapkota (2020)
Anxiety	Mantler (2022), Sapkota (2020), Sapkota (2022)
Distress	Abrahams (2022), Ammerman (2022), Curry (2006)
Maternal Physical Health/Functioning & Maternal Nutrition	Belaid (2021), Briaux (2020), Cripe (2010), Fergusson (2005), Flaathen (2022), Jack (2019), Tiwari (2005)
Quality of Life	Alizadeh (2021), Cripe (2010), Dinmohammadi (2021), Flaathen (2022), Jack (2019), Poleschuk (2019), Sapkota (2020), Sapkota (2022), Taft (2011), Tiwari (2005), Trabold (2020), Van Parys (2017)
Social Support	Rastegar (2021), Sapkota (2020), Sapkota (2022), Taft (2011)
Maternal Obstetrical Health	
Miscarriage or Stillbirth	Arora (2019), Olds (2004), Olds (2007), Subramanian (2012)
Preterm Birth	Arora (2019), Kiely (2010), Olds (2004), Subramanian (2012)
Hospitalization During Pregnancy	Subramanian (2012)
Gestational Hypertension or Diabetes	Subramanian (2012)
Unintended Pregnancy	Miller (2016)
Late Entry into Prenatal Care	Briaux (2020)
Neonatal & Child Health Outcomes	
Low Birthweight/Small for Gestational Age	Briaux (2020), Jacobs (2016), Kiely (2010), Olds (2004), Olds (2007), Subramanian (2012)
Perinatal Death	Subramanian (2012)
Birth Outcome, General	Kramer (2012)
Failed Newborn to Mother Bonding	Arora (2019), Jackson (2020), Khalili (2020), Mantler (2022), Rowe (2014), Taft (2011)
NICU Admission	Olds (2004), Subramanian (2012)
Child Feeding, Growth & Medical Care	Barrington (2022), Belaid (2021), Briaux (2020), Fergusson (2005)
Parenting Outcomes & Family‐Related Measures	
Child Abuse	Barlow (2007), Fergusson (2005), Fergusson (2013), McIntosh (2009)
Infant or Child Behavioral Adjustment & Functioning	Barlow (2007), Fergusson (2005), Fergusson (2013), Jacobs (2016)
Parenting Competence and Distress	Ammerman (2022), Belaid (2021), Duggan (2004), Fergusson (2005), Fergusson (2013), Jacobs (2016), Kan (2015)
Family Functioning	Akor (2019)
Poverty Measures	Barrington (2022), Belaid (2021), Briaux (2020), Curry (2006), Fergusson (2005), Olds (2004), Olds (2007), Olds (2010), Peterman (2022)
Awareness of Violence, IPV Services & Sense of Self	
Awareness & Knowledge of Abuse	Arora (2019), Bacchus (2016), Domoney (2019), Haberland (2020), Kan (2021), Miller (2016), Rastegar (2021), Turan (2013)
Awareness of IPV Services	Kan (2021); Miller (2011), Taft (2015)
Seeking Help/Making a Change	Arora (2019), Bacchus (2010), Belaid (2021), Cripe (2010), Duggan (2004), Flaathen (2022), Haberland (2020), Kan (2021), Kramer (2012), Laughon (2011), McFarlane (2000), Miller (2011), Miller (2016), Poleschuk (2019), Sapkota (2020), Sapkota (2022), Taft (2015), Van Parys (2017)
Sense of Self	Bacchus (2016), Bahadir‐Yilmaz (2018), Barrington (2022), Briaux (2020), Domoney (2019), Duggan (2004), Kan (2021), Kramer (2012), Mantler (2022), Olds), (2004), Sapkota (2022), Trabold (2020), Van Parys (2017)
Acceptability & Feasibility	
Acceptability	Abrahams (2022), Bacchus (2016), Haberland (2020), Jackson (2020), Katz (2008), Kramer (2012), Rowe (2014), Sapkota (2020), Taft (2011), Trabold (2020), Van Parys (2017), Walter (2021), Zlotnick (2011), Zlotnick (2019)
Feasibility	Katz (2008), McConnell (2020), Sapkota (2022), Trabold (2020)
Cost‐Effectiveness	McIntosh (2009)

Of the 67 studies, most (*n* = 38) measured intervention effect on IPV revictimization. Forms of IPV revictimization included physical, psychological, and sexual IPV. A total of 26 studies evaluated intervention effect on maternal mental health. Measured outcomes related to maternal mental health included depression, PTSD, substance use, anxiety, general distress, and social support. Fifteen studies measured maternal physical health outcomes, including general physical health, physical functioning, quality of life, and maternal nutrition.

Eight studies measured maternal obstetrical health outcomes. Outcomes included unintended pregnancy, late entry into prenatal care, gestational diabetes, gestational hypertension, hospitalization during pregnancy, miscarriage, stillbirth, and preterm birth.

Neonatal and child health outcomes were measured in 18 studies. Neonatal health outcomes included birth outcome, low birthweight, small for gestational age, neonatal intensive care unit admission, perinatal death, and failed newborn to mother bonding. Child health outcomes included feeding, growth, and medical care.

A total of 17 studies measured parenting and family‐related outcomes. These included infant and child behavioral adjustment and functioning, family functioning, parenting competence, parenting distress, and child abuse. Additionally, poverty measures were included, such as household food insecurity, household per capita expenditures, financial stress, and environmental hygiene.

Outcomes related to awareness of IPV and available services, actions to reduce violence, and self‐esteem were included in 28 studies. Measured outcomes included awareness and knowledge of IPV and awareness of IPV services available in the community. Additionally, actions to seek help or reduce violence were measured (e.g., safety behaviors, safety planning, use of IPV services, leaving abusive partner) as were outcomes related to self‐esteem (e.g., readiness to change, empowerment, self‐efficacy, coping mechanisms, learned helplessness, resourcefulness, desire for IPV services, and acceptance of IPV).

Finally, 17 studies evaluated intervention acceptability and/or feasibility. Examples of acceptability measures include perception of care provided during the intervention, and feelings of support during the intervention. Cost‐effectiveness measures were also included in one study (McIntosh et al., 2009).

### 5.5 Study quality/risk of bias in included studies

We did not perform assessment of study quality/risk of bias as part of this scoping review.

### 5.6 Effects of interventions

**Figure 3:**
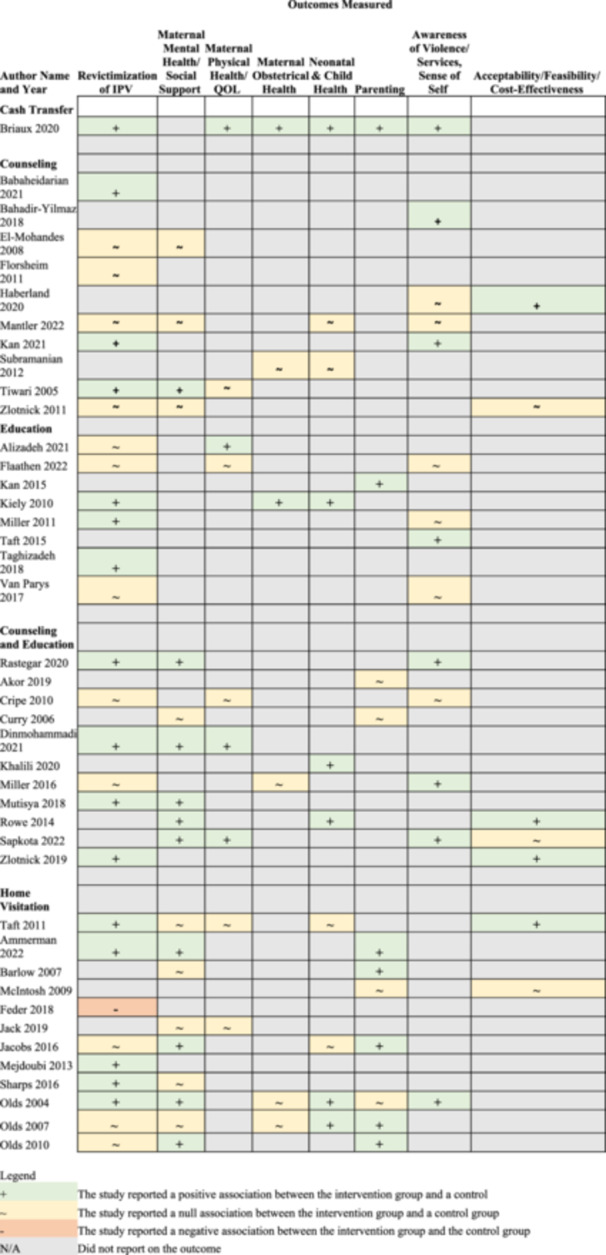
Evidence map of the effect of perinatal intervention type by outcome as measured in 42 studies. NB: As this is a scoping review, the study effect sizes are not presented. This is a narrative categorization based on the conclusions of the study and the published effects. Additionally, results should be interpreted with caution as risk of bias was not assessed.

We did not assess the effects of interventions as part of this scoping review. However, we did create a narrative synthesis of the effects of interventions based on the conclusions of the individual studies and the published effects (Figure 3). Importantly, this synthesis of effects should be interpreted with caution as we did not assess the risk of bias of individual studies.

## 6 DISCUSSION

### 6.1 Summary of main results

A range of interventions supporting survivors exposed to IPV during the perinatal period exist. These interventions have been implemented across the world, in a variety of settings including hospital and community‐based settings. Study populations include prenatal and post‐partum women experiencing IPV, and many studies restricted their study sample based on various sociodemographic factors including socioeconomic status, income, age, rural versus urban setting, ethnicity, and mental health concerns. Comparators most often received either normal prenatal/post‐partum care or additional supports (i.e., information on community organizations that provide help to survivors of IPV, referral to mental health services).

Existing interventions are diverse, with most incorporating counseling or education, and others using a home visitation or cash transfer format. Interventions using a cash transfer format occurred in lower‐middle income countries such as Ghana and Togo. Meanwhile, home visitation programs occurred in higher income countries such as the United States, United Kingdom, Netherlands, Australia, and New Zealand. However, home visitation programs often targeted vulnerable groups, such as low‐income women. Counseling and education interventions occurred in both lower‐middle income and higher income countries. Most often, these interventions are designed solely to target IPV and its associated outcomes. However, sometimes broad interventions are designed to target many psychosocial elements including IPV.

The measured outcomes in included studies covered a wide range, including IPV revictimization, maternal mental health, maternal physical health, maternal obstetrical health, neonatal and child health, parenting and family‐related measures, awareness of abuse and community IPV services, actions to reduce violence, and acceptability and feasibility of the intervention. Most studies measured intervention effect on IPV revictimization. Additionally, many studies measured intervention effect on an aspect of maternal mental health, and many studies assessed survivor knowledge or acceptance of violence, knowledge of resources available to them, or actions to reduce violence. Meanwhile, only 18 studies measured intervention effect on neonatal or child health outcomes, and even fewer studies assessed intervention acceptability of feasibility (*n* = 17) and (*n* = 8) intervention effect on maternal obstetrical outcomes.

### 6.2 Overall completeness and applicability of evidence

Not applicable to this review.

### 6.3 Quality of the evidence

Not applicable to this review.

### 6.4 Potential biases in the review process

Studies were only eligible for inclusion if published in English or French and after 2000. Many articles were excluded because they solely interviewed providers about their experience delivering the intervention. These articles may have included unique interventions that were not otherwise included in this review and may have provided relevant knowledge about the range of existing interventions for IPV during the perinatal period. Bias could have been introduced when the research team had different interpretation of the inclusion criteria. Therefore, all full‐text review decisions were made by two researchers, with conflicts resolved by a third researcher.

### 6.5 Agreements and disagreements with other studies or reviews

The findings of this scoping review are consistent with the existing literature. Specifically, the high frequency of IPV revictimization as a measured outcome is reflected in existing studies. One systematic review evaluating existing screening procedures and interventions for women experiencing IPV, limited to pregnancy, included four intervention studies (O'Reilly et al., 2010). All four studies evaluated intervention effect on IPV revictimization, however no other outcomes (e.g., maternal or neonatal health, acceptability and feasibility, etc.) were assessed. In this study, O'Reilly et al. identified there is some evidence that interventions reduced IPV revictimization, however the evidence was limited due to the small number of studies and sample sizes.

Similarly, one systematic review evaluating interventions for IPV around the time of pregnancy, limited to RCTs, primarily reported on IPV revictimization (Van Parys et al., 2014). Other outcomes were included, such as maternal physical and/or mental health, quality of life, subsequent miscarriages, low birth weight/prematurity, safety behaviors, and help seeking behavior, however they were reported in fewer articles (Van Parys et al., 2014). Researchers found that home visitation and counseling programs produced promising results, as five studies reported a significant decrease in IPV revictimization (Van Parys et al., 2014). However, limited evidence was found for improved maternal mental health, quality of life, subsequent miscarriages, and decreased low birth weight/prematurity (Van Parys et al., 2014).

Another systematic review conducted by Jahanfar et al. in 2014 also found that obstetrical, neonatal, and child health outcomes are infrequently measured. Only one study reported findings for neonatal outcomes (e.g., preterm delivery, low birth weight), and there were no significant differences found (Jahanfar et al., 2014). Additionally, no studies reported results for many other neonatal and obstetrical outcomes such as maternal mortality, antepartum hemorrhage, placental abruption, stillbirth, or miscarriage. While this systematic review primarily reported on IPV revictimization, there was limited evidence for reduction of physical, sexual, or psychological IPV revictimization during pregnancy and up to 1 year post‐partum (Jahanfar et al., 2014). These findings confirm that a knowledge gap in the current literature is the lack of studies measuring intervention effect on obstetrical, neonatal, and child health outcomes.

Previous studies that have described existing IPV interventions have reported counseling as a common intervention type. A 2020 systematic review by Daley et al. describing IPV interventions in low‐ and middle‐income countries included a total of six studies (Daley et al., 2020). Of these, five evaluated counseling interventions, and one intervention consisted solely of supported referrals to community resources (Daley et al., 2020). In this study, researchers found that counseling interventions resulted in a significant decrease in IPV as well as a significant increase in family support (Daley et al., 2020). Additionally, a 2021 systematic review by Reyes et al. included a total of 31 studies and demonstrated that most IPV interventions that included counseling resulted in decreased IPV revictimization and severity (Reyes et al., 2021).

Other existing reviews have often narrowed the scope of included interventions, such as the 2023 thematic review by Adams et al. that explored the role of nurses in home visiting programs for those experiencing family violence (Adams et al., 2023). This study identified the importance of building a strong relationship with survivors and provided insight into the challenges nurses face in the identification of violence and during safety planning discussions (Adams et al., 2023). Meanwhile, Rivas et al., 2019 solely assessed advocacy interventions for IPV, and evaluated these interventions in terms of which interventions are effective in which contexts. They found that while advocacy interventions often benefit IPV survivors, when IPV is severe, interventions may increase violence (Rivas et al., 2019).

### 6.6 Limitations

One limitation of this scoping review is that we excluded interventions targeted solely at healthcare providers. These interventions may have included unique components that were otherwise not included in this review. Similarly, interventions designed to act as primary IPV prevention were excluded and may have included valuable information on existing perinatal IPV interventions. The second limitation of our scoping review is the lack of evaluation of intervention effectiveness to further understand which interventions are most successful in reducing IPV victimization and its associated adverse outcomes. Providing this information would add important context, and we hope this can be addressed in future studies. Lastly, we did not assess risk of bias of included studies.

## 7 AUTHORS' CONCLUSIONS

### 7.1 Implications for practice

Based on the findings of this scoping review, the existing interventions for IPV during the perinatal period are diverse with regard to setting, content, and resources required for their implementation. To date, perinatal IPV interventions have been evaluated in survivors' homes, in community‐based settings, and in hospital‐based settings. Additionally, at least nine RCTs have been evaluated in each setting to assess intervention effectiveness. This provides evidence that the many barriers to aiding IPV victims, such as the myth that IPV is nearly impossible to identify and intervene, are not founded. Rather, identifying IPV during the perinatal period and intervening is possible, regardless of the environment in which the healthcare provider and survivor are interacting.

As mentioned previously, screening for IPV without providing adequate follow‐up is considered unethical as per the World Health Organization. It is critical to provide an adequate intervention to survivors following screening, and this review provides 57 unique interventions that do so. As this is a scoping review, we recognize that we are unable to comment on the effectiveness of the interventions, however we have consolidated information on 67 studies from around the world designed to improve outcomes for perinatal IPV survivors.

### 7.2 Implications for research

IPV survivors are a vulnerable population in need of assistance from those who can provide it. As healthcare providers, we are in a unique position to provide help to survivors, especially during the perinatal period. There is a critical need to continue designing and implementing interventions to reduce exposure to violence for both the mother and infant to improve outcomes for all.

As the adverse effects of IPV during the perinatal period on obstetrical and neonatal health are well documented, it is imperative that interventions designed to target perinatal IPV be effective at mitigating these adverse effects. Further research must evaluate existing perinatal IPV intervention effect on obstetrical outcomes and neonatal and child health outcomes. Obtaining permission to link to clinical records or engaging survivors in the research process (e.g., via interviews) are effective ways to do so.

## CONTRIBUTIONS OF AUTHORS

KM conceptualized and designed the study. YF, KF, and KM designed the search strategy and KF conducted the search. OM, YF, RF, AL, and KM conducted the article screening, and OM, YF, RF, and AL performed the data extraction. OM drafted the manuscript and KM critically edited and reviewed the manuscript. KM has primary responsibility for the final content. All authors read and approved the final manuscript.

## DECLARATIONS OF INTEREST

The authors have no interests to declare.

## SOURCES OF SUPPORT

Internal sources: No sources of support provided.

External sources: No sources of support provided.

## Supporting information

Supporting information.
