# Supplementary material for: Interventions for intimate partner violence during the perinatal period: A scoping review: A systematic review
Source: Campbell Syst Rev. 2024 Jul 15;20(3):e1423. doi: 10.1002/cl2.1423 (PMC11247475; doi:10.1002/cl2.1423)
Supplement: Supplementary file 1 — Supporting information. [file CL2-20-e1423-s001.docx]

**Appendix A: Complete Search Strategy**

Date Conducted: June 1, 2022

Search Strategies and Method Section

| Ovid MEDLINE(R) ALL <1946 to May 31, 2022> | | |
| --- | --- | --- |
|  |  |  |
| 1 | exp perinatal care/ or preconception care/ or prenatal care/ or Maternal Health Services/ or Maternal Health/ or Maternal Welfare/ | 61142 |
| 2 | exp Pregnancy/ | 969564 |
| 3 | exp Pregnancy Complications/ | 460115 |
| 4 | peripartum period/ or exp postpartum period/ or exp pregnancy trimesters/ | 116147 |
| 5 | Pregnant Women/ | 12281 |
| 6 | Obstetrics/ | 24062 |
| 7 | exp Breast Feeding/ or Bottle Feeding/ | 43520 |
| 8 | (maternal* or pregnan* or gestation*).ti,ab,kf. | 830487 |
| 9 | (expect* adj3 (mother* or mom? or child* or baby or babies)).ti,ab,kf. | 5807 |
| 10 | (prenatal* or perinatal* or antenatal* or postnatal* or postpartum* or post-partum* or pre-partum*).ti,ab,kf. | 366556 |
| 11 | (Peripartum* or puerperium* or puerperal*).ti,ab,kf. | 20560 |
| 12 | Obstetric*.ti,ab,kf. | 111815 |
| 13 | Parturition*.ti,ab,kf. | 15844 |
| 14 | (Lactat* or breastfeed* or breast feed* or breast milk or milk expression* or ((bottle* or formula*) adj3 feed*)).ti,ab,kf. | 229449 |
| 15 | (Childbirth* or child-bearing).ti,ab,kf. | 24545 |
| 16 | ((Child* or newborn* or infant*) adj3 birth*).ti,ab,kf. | 47299 |
| 17 | birthing.ti,ab,kf. | 2439 |
| 18 | ((before or after) adj3 birth*).ti,ab,kf. | 61364 |
| 19 | (pre-birth* or post-birth*).ti,ab,kf. | 801 |
| 20 | (prepregnancy or pre-pregnancy or post-pregnancy or postpregnancy or preconception* or pre-conception* or postconception* or post-conception* or periconception* or peri-conception*).ti,ab,kf. | 25383 |
| 21 | or/1-20 | 1646795 |
| 22 | Battered Women/ | 2703 |
| 23 | domestic violence/ or spouse abuse/ or gender-based violence/ or intimate partner violence/ | 18734 |
| 24 | ((wife or wives or wom#n) adj3 batter*).ti,ab,kf. | 1009 |
| 25 | ((violen* or abus*) adj3 (partner* or wom#n or spous* or wife or wives or marital or marriage*)).ti,ab,kf. | 18748 |
| 26 | ((domestic* or home*) adj3 (violen* or abus*)).ti,ab,kf. | 10315 |
| 27 | ((relation* or interperson*) adj3 (abuse* or violen*)).ti,ab,kf. | 6750 |
| 28 | (violen* adj3 (date* or dating)).ti,ab,kf. | 1498 |
| 29 | (date* adj3 rape*).ti,ab,kf. | 281 |
| 30 | ((domestic* or marital or partner* or spous*) adj3 rape*).ti,ab,kf. | 205 |
| 31 | ((domestic* or marital or partner* or spous*) adj3 (sex* adj1 (abuse* or assault*))).ti,ab,kf. | 492 |
| 32 | (gender-based adj3 (violen* or abus*)).ti,ab,kf. | 1464 |
| 33 | or/22-32 | 35800 |
| 34 | 21 and 33 | 5763 |
| 35 | (comment or editorial or letter).pt. | 2065330 |
| 36 | 34 not 35 | 5628 |
| 37 | limit 36 to yr="2000-current" | 4850 |

| Ovid Embase <1947 to 2022 May 31> | | |
| --- | --- | --- |
|  |  |  |
| 1 | exp pregnancy/ | 866831 |
| 2 | exp pregnancy disorder/ | 680421 |
| 3 | exp breast feeding/ or exp breast feeding education/ | 64549 |
| 4 | exp childbirth/ or exp childbirth education/ | 70964 |
| 5 | obstetric procedure/ or exp perinatal care/ or exp postnatal care/ or exp prenatal care/ or prepregnancy care/ | 350881 |
| 6 | exp obstetrics/ | 46141 |
| 7 | maternal health service/ | 2422 |
| 8 | exp maternal care/ | 52309 |
| 9 | perinatal period/ | 39477 |
| 10 | pregnant woman/ | 105268 |
| 11 | exp bottle feeding/ | 4310 |
| 12 | (maternal* or pregnan* or gestation*).ti,ab,kw. | 1118438 |
| 13 | (expect* adj3 (mother* or mom? or child* or baby or babies)).ti,ab,kw. | 7856 |
| 14 | (prenatal* or perinatal* or antenatal* or postnatal* or postpartum* or post-partum* or pre-partum*).ti,ab,kw. | 493232 |
| 15 | (Peripartum* or puerperium* or puerperal*).ti,ab,kw. | 25335 |
| 16 | Obstetric*.ti,ab,kw. | 168753 |
| 17 | Parturition*.ti,ab,kw. | 20600 |
| 18 | (Lactat* or breastfeed* or breast feed* or breast milk or milk expression* or ((bottle* or formula*) adj3 feed*)).ti,ab,kw. | 291715 |
| 19 | (Childbirth* or child-bearing).ti,ab,kw. | 32128 |
| 20 | ((Child* or newborn* or infant*) adj3 birth*).ti,ab,kw. | 64230 |
| 21 | birthing.ti,ab,kw. | 2903 |
| 22 | ((before or after) adj3 birth*).ti,ab,kw. | 87918 |
| 23 | (pre-birth* or post-birth*).ti,ab,kw. | 1183 |
| 24 | (prepregnancy or pre-pregnancy or post-pregnancy or postpregnancy or preconception* or pre-conception* or postconception* or post-conception* or periconception* or peri-conception*).ti,ab,kw. | 29275 |
| 25 | or/1-24 | 2174382 |
| 26 | Battered Women/ or domestic violence/ or partner violence/ | 25597 |
| 27 | gender based violence/ | 1236 |
| 28 | ((wife or wives or wom#n) adj3 batter*).ti,ab,kw. | 1044 |
| 29 | ((violen* or abus*) adj3 (partner* or wom#n or spous* or wife or wives or marital or marriage*)).ti,ab,kw. | 20516 |
| 30 | ((domestic* or home*) adj3 (violen* or abus*)).ti,ab,kw. | 11090 |
| 31 | ((relation* or interperson*) adj3 (abuse* or violen*)).ti,ab,kw. | 7979 |
| 32 | (violen* adj3 (date* or dating)).ti,ab,kw. | 1454 |
| 33 | (date* adj3 rape*).ti,ab,kw. | 323 |
| 34 | ((domestic* or marital or partner* or spous*) adj3 rape*).ti,ab,kw. | 252 |
| 35 | ((domestic* or marital or partner* or spous*) adj3 (sex* adj1 (abuse* or assault*))).ti,ab,kw. | 635 |
| 36 | (gender-based adj3 (violen* or abus*)).ti,ab,kw. | 1376 |
| 37 | or/26-36 | 42754 |
| 38 | 25 and 37 | 7424 |
| 39 | letter/ | 1152721 |
| 40 | (Conference Abstract or Editorial or Letter).pt. | 6368578 |
| 41 | or/39-40 | 6374587 |
| 42 | 38 not 41 | 6259 |
| 43 | limit 42 to yr="2000 -Current" | 5568 |

| Ovid Cochrane Central Register of Controlled Trials <April 2022> | | |
| --- | --- | --- |
|  |  |  |
| 1 | exp Pregnancy/ | 23656 |
| 2 | exp pregnancy complications/ | 12581 |
| 3 | exp prenatal diagnosis/ | 851 |
| 4 | exp maternal health services/ | 2444 |
| 5 | peripartum period/ or exp postpartum period/ | 1851 |
| 6 | exp lactation/ or parity/ | 1561 |
| 7 | exp Perinatal Care/ | 604 |
| 8 | obstetrics/ | 208 |
| 9 | Obstetric Nursing/ | 45 |
| 10 | bottle feeding/ or exp breast feeding/ | 2148 |
| 11 | milk, human/ | 1098 |
| 12 | maternal welfare/ | 106 |
| 13 | maternal health/ | 75 |
| 14 | exp Pregnancy Trimesters/ | 1822 |
| 15 | pregnant women/ | 449 |
| 16 | (maternal* or pregnan* or gestation*).tw. | 81523 |
| 17 | (expect* adj3 (mother* or mom? or child* or baby or babies)).tw. | 621 |
| 18 | (prenatal* or perinatal* or antenatal* or postnatal* or postpartum* or post-partum* or pre-partum*).tw. | 25169 |
| 19 | (Peripartum* or puerperium* or puerperal*).tw. | 1393 |
| 20 | Obstetric*.tw. | 10027 |
| 21 | Parturition*.tw. | 239 |
| 22 | (Lactat* or breastfeed* or breast feed* or breast milk or milk expression* or ((bottle* or formula*) adj3 feed*)).tw. | 24202 |
| 23 | (Childbirth* or child-bearing).tw. | 6045 |
| 24 | ((Child* or newborn* or infant*) adj3 birth*).tw. | 6434 |
| 25 | birthing.tw. | 205 |
| 26 | ((before or after) adj3 birth*).tw. | 5502 |
| 27 | (pre-birth* or post-birth*).tw. | 160 |
| 28 | (prepregnancy or pre-pregnancy or post-pregnancy or postpregnancy or preconception* or pre-conception* or postconception* or post-conception* or periconception* or peri-conception*).tw. | 1822 |
| 29 | or/1-28 | 117233 |
| 30 | battered women/ | 67 |
| 31 | domestic violence/ or spouse abuse/ | 347 |
| 32 | gender-based violence/ or intimate partner violence/ | 241 |
| 33 | ((wife or wives or wom#n) adj3 batter*).tw. | 44 |
| 34 | ((violen* or abus*) adj3 (partner* or wom#n or spous* or wife or wives or marital or marriage*)).tw. | 1447 |
| 35 | ((domestic* or home*) adj3 (violen* or abus*)).tw. | 544 |
| 36 | ((relation* or interperson*) adj3 (abuse* or violen*)).tw. | 358 |
| 37 | (violen* adj3 (date* or dating)).tw. | 141 |
| 38 | (date* adj3 rape*).tw. | 27 |
| 39 | ((domestic* or marital or partner* or spous*) adj3 rape*).tw. | 9 |
| 40 | ((domestic* or marital or partner* or spous*) adj3 (sex* adj1 (abuse* or assault*))).tw. | 31 |
| 41 | (gender-based adj3 (violen* or abus*)).tw. | 107 |
| 42 | or/30-41 | 2341 |
| 43 | 29 and 42 | 474 |
| 44 | limit 43 to yr="2000 -Current" | 456 |

| Ovid APA PsycInfo <1806 to May Week 4 2022> | | |
| --- | --- | --- |
|  |  |  |
| 1 | perinatal period/ or antepartum period/ or intrapartum period/ or postnatal period/ or exp pregnancy/ | 52393 |
| 2 | exp prenatal care/ or exp obstetrics/ or prenatal diagnosis/ | 6054 |
| 3 | pregnancy outcomes/ | 1303 |
| 4 | obstetrics/ or childbirth training/ | 1596 |
| 5 | breast feeding/ or lactation/ | 5340 |
| 6 | bottle feeding/ | 273 |
| 7 | (maternal* or pregnan* or gestation*).tw. | 109503 |
| 8 | (expect* adj3 (mother* or mom? or child* or baby or babies)).tw. | 6251 |
| 9 | (prenatal* or perinatal* or antenatal* or postnatal* or postpartum* or post-partum* or pre-partum*).tw. | 60746 |
| 10 | (Peripartum* or puerperium* or puerperal*).tw. | 1301 |
| 11 | Obstetric*.tw. | 6833 |
| 12 | Parturition*.tw. | 1405 |
| 13 | (Lactat* or breastfeed* or breast feed* or breast milk or milk expression* or ((bottle* or formula*) adj3 feed*)).tw. | 12642 |
| 14 | (Childbirth* or child-bearing).tw. | 6523 |
| 15 | ((Child* or newborn* or infant*) adj3 birth*).tw. | 10588 |
| 16 | birthing.tw. | 669 |
| 17 | ((before or after) adj3 birth*).tw. | 8563 |
| 18 | (pre-birth* or post-birth*).tw. | 354 |
| 19 | (prepregnancy or pre-pregnancy or post-pregnancy or postpregnancy or preconception* or pre-conception* or postconception* or post-conception* or periconception* or peri-conception*).tw. | 3717 |
| 20 | or/1-19 | 171971 |
| 21 | battered females/ or domestic violence/ or intimate partner violence/ | 24319 |
| 22 | ((wife or wives or wom#n) adj3 batter*).tw. | 2612 |
| 23 | ((violen* or abus*) adj3 (partner* or wom#n or spous* or wife or wives or marital or marriage*)).tw. | 24041 |
| 24 | ((domestic* or home*) adj3 (violen* or abus*)).tw. | 15016 |
| 25 | ((relation* or interperson*) adj3 (abuse* or violen*)).tw. | 11102 |
| 26 | (violen* adj3 (date* or dating)).tw. | 2157 |
| 27 | (date* adj3 rape*).tw. | 495 |
| 28 | ((domestic* or marital or partner* or spous*) adj3 rape*).tw. | 427 |
| 29 | ((domestic* or marital or partner* or spous*) adj3 (sex* adj1 (abuse* or assault*))).tw. | 862 |
| 30 | (gender-based adj3 (violen* or abus*)).tw. | 1152 |
| 31 | or/21-30 | 46446 |
| 32 | 20 and 31 | 3567 |
| 33 | (Non-Peer-Reviewed Journal or Book or Authored Book or Edited Book).pt. | 516936 |
| 34 | 32 not 33 | 3188 |
| 35 | limit 34 to yr="2000 -Current" | 2814 |

CINAHL (EBSCOHost)

| **#** | **Query** | **Results** |
| --- | --- | --- |
| S1 | (MH "Maternal-Child Care+") | 63,326 |
| S2 | (MH "Pregnancy+") OR (MH "Pregnancy, Multiple+") OR (MH "Pregnancy Trimesters+") OR (MH "Prenatal Nutritional Physiology") OR (MH "Postnatal Period+") OR (MH "Periconceptual Period") | 242,739 |
| S3 | (MH "Pregnancy Complications+") | 104,417 |
| S4 | (MH "Breast Feeding+") OR (MH "Bottle Feeding") OR (MH "Infant Feeding") OR (MH "Infant Feeding Schedules") OR (MH "Infant Feeding, Supplemental") | 29,779 |
| S5 | (MH "Obstetric Emergencies") | 833 |
| S6 | (MH "Obstetric Nursing") OR (MH "Perinatal Nursing") | 4,963 |
| S7 | (MH "Surgery, Obstetrical+") | 34,663 |
| S8 | (MH "Diagnosis, Obstetric+") | 26,255 |
| S9 | TI ( maternal* or pregnan* or gestation* or prenatal* or perinatal* or antenatal* or postnatal* or postpartum* or post-partum* or pre-partum* or Peripartum* or puerperium* or puerperal* or Obstetric* or Parturition* or Childbirth* or "child-bearing" or birthing or "pre-birth*" or "post-birth*" or prepregnancy or "pre-pregnancy" or "post-pregnancy" or postpregnancy or preconception* or "pre-conception*" or postconception* or "post-conception*" or periconception* or "peri-conception*" ) OR AB ( maternal* or pregnan* or gestation* or prenatal* or perinatal* or antenatal* or postnatal* or postpartum* or post-partum* or pre-partum* or Peripartum* or puerperium* or puerperal* or Obstetric* or Parturition* or Childbirth* or "child-bearing" or birthing or "pre-birth*" or "post-birth*" or prepregnancy or "pre-pregnancy" or "post-pregnancy" or postpregnancy or preconception* or "pre-conception*" or postconception* or "post-conception*" or periconception* or "peri-conception*" ) | 296,080 |
| S10 | TI ( (expect* N3 (mother* or mom? or child* or baby or babies)) ) OR AB ( (expect* N3 (mother* or mom? or child* or baby or babies)) ) | 4,331 |
| S11 | TI ( (Lactat* or breastfeed* or breast feed* or breast milk or milk expression* or ((bottle* or formula*) N3 feed*)) ) OR AB ( (Lactat* or breastfeed* or breast feed* or breast milk or milk expression* or ((bottle* or formula*) N3 feed*)) ) | 46,491 |
| S12 | TI ( ((Child* or newborn* or infant*) N3 birth*) ) OR AB ( ((Child* or newborn* or infant*) N3 birth*) ) | 21,575 |
| S13 | TI ( ((before or after) N3 birth*) ) OR AB ( ((before or after) N3 birth*) ) | 14,905 |
| S14 | S1 OR S2 OR S3 OR S4 OR S5 OR S6 OR S7 OR S8 OR S9 OR S10 OR S11 OR S12 OR S13 | 438,417 |
| S15 | (MH "Battered Women") | 4,134 |
| S16 | (MH "Domestic Violence") OR (MH "Intimate Partner Violence") OR (MH "Gender-Based Violence") | 20,639 |
| S17 | (MH "Dating Violence") | 890 |
| S18 | TI ( ((wife or wives or wom?n) N3 batter*) ) OR AB ( ((wife or wives or wom?n) N3 batter*) ) | 659 |
| S19 | TI ( ((violen* or abus*) N3 (partner* or wom?n or spous* or wife or wives or marital or marriage*)) ) OR AB ( ((violen* or abus*) N3 (partner* or wom?n or spous* or wife or wives or marital or marriage*)) ) | 16,027 |
| S20 | TI ( ((domestic* or home*) N3 (violen* or abus*)) ) OR AB ( ((domestic* or home*) N3 (violen* or abus*)) ) | 8,682 |
| S21 | TI ( ((relation* or interperson*) N3 (abuse* or violen*)) ) OR AB ( ((relation* or interperson*) N3 (abuse* or violen*)) ) | 5,468 |
| S22 | TI ( (violen* N3 (date* or dating)) ) OR AB ( (violen* N3 (date* or dating)) ) | 1,298 |
| S23 | TI (date* N3 rape*) OR AB (date* N3 rape*) | 106 |
| S24 | TI ( ((domestic* or marital or partner* or spous*) N3 rape*) ) OR AB ( ((domestic* or marital or partner* or spous*) N3 rape*) ) | 146 |
| S25 | TI ( ((domestic* or marital or partner* or spous*) N3 (sex* N1 (abuse* or assault*))) ) OR AB ( ((domestic* or marital or partner* or spous*) N3 (sex* N1 (abuse* or assault*))) ) | 576 |
| S26 | TI ( (gender-based N3 (violen* or abus*)) ) OR AB ( (gender-based N3 (violen* or abus*)) ) | 888 |
| S27 | S15 OR S16 OR S17 OR S18 OR S19 OR S20 OR S21 OR S22 OR S23 OR S24 OR S25 OR S26 | 32,729 |
| S28 | S14 AND S27 | 4,527 |
| S29 | S14 AND S27  Limiters - Published Date: 20000101-20221231  Source Types: Academic Journals | 3,929 |
| S30 | (ZT "book") or (ZT "book chapter") or (ZT "book review") or (ZT "commentary") or (ZT "editorial") ") or (ZT "letter") or (ZT "letter to the editor") or (ZT "opinion") | 427,118 |
| S31 | S29 NOT S30 | 3,846 |

**Web of Science Core Collection**

(maternal* or pregnan* or gestation* or prenatal* or perinatal* or antenatal* or postnatal* or postpartum* or "post-partum*" or "pre-partum*" or Peripartum* or puerperium* or puerperal* or Obstetric* or Parturition* or Lactat* or breastfeed* or breast feed* or milk expression* or ((bottle* or formula*) NEAR/3 feed*) or Childbirth* or "child-bearing" or birthing or ((Child* or newborn* or infant*) NEAR/3 birth*) or ((before or after) NEAR/3 birth*) or "pre-birth*" or "post-birth*" or prepregnancy or "pre-pregnancy" or "post-pregnancy" or postpregnancy or preconception* or "pre-conception*" or postconception* or "post-conception*" or periconception* or "peri-conception*" or (expect* NEAR/3 (mother* or mom? or child* or baby or babies))) AND (((wife or wives or wom?n) NEAR/3 batter*) or ((violen* or abus*) NEAR/3 (partner* or wom?n or spous* or wife or wives or marital or marriage*)) or ((domestic* or home*) NEAR/3 (violen* or abus*)) or ((relation* or interperson*) NEAR/3 (abuse* or violen*)) or (violen* NEAR/3 (date* or dating)) or (date* NEAR/3 rape*) or ((domestic* or marital or partner* or spous*) NEAR/3 rape*) or ((domestic* or marital or partner* or spous*) NEAR/3 (sex* NEAR/1 (abuse* or assault*))) or (gender-based NEAR/3 (violen* or abus*))) (Title) or (maternal* or pregnan* or gestation* or prenatal* or perinatal* or antenatal* or postnatal* or postpartum* or "post-partum*" or "pre-partum*" or Peripartum* or puerperium* or puerperal* or Obstetric* or Parturition* or Lactat* or breastfeed* or breast feed* or milk expression* or ((bottle* or formula*) NEAR/3 feed*) or Childbirth* or "child-bearing" or birthing or ((Child* or newborn* or infant*) NEAR/3 birth*) or ((before or after) NEAR/3 birth*) or "pre-birth*" or "post-birth*" or prepregnancy or "pre-pregnancy" or "post-pregnancy" or postpregnancy or preconception* or "pre-conception*" or postconception* or "post-conception*" or periconception* or "peri-conception*" or (expect* NEAR/3 (mother* or mom? or child* or baby or babies))) AND (((wife or wives or wom?n) NEAR/3 batter*) or ((violen* or abus*) NEAR/3 (partner* or wom?n or spous* or wife or wives or marital or marriage*)) or ((domestic* or home*) NEAR/3 (violen* or abus*)) or ((relation* or interperson*) NEAR/3 (abuse* or violen*)) or (violen* NEAR/3 (date* or dating)) or (date* NEAR/3 rape*) or ((domestic* or marital or partner* or spous*) NEAR/3 rape*) or ((domestic* or marital or partner* or spous*) NEAR/3 (sex* NEAR/1 (abuse* or assault*))) or (gender-based NEAR/3 (violen* or abus*))) (Abstract) and 2000 or 2002 or 2001 or 2003 or 2004 or 2005 or 2006 or 2007 or 2008 or 2009 or 2022 or 2021 or 2020 or 2019 or 2018 or 2017 or 2016 or 2015 or 2014 or 2013 or 2012 (Publication Years) and Meeting Abstracts or Editorial Materials or Letters or Book Reviews or News Items or Book Chapters (Exclude – Document Types)

Results: 4,563

**Applied Social Sciences Index & Abstracts (ProQuest)**

ab(((wife OR wives OR wom?n) NEAR/3 batter*) OR ((violen* OR abus*) NEAR/3 (partner* OR wom?n OR spous* OR wife OR wives OR marital OR marriage*)) OR ((domestic* OR home*) NEAR/3 (violen* OR abus*)) OR ((relation* OR interperson*) NEAR/3 (abuse* OR violen*)) OR (violen* NEAR/3 (date* OR dating)) OR (date* NEAR/3 rape*) OR ((domestic* OR marital OR partner* OR spous*) NEAR/3 rape*) OR ((domestic* OR marital OR partner* OR spous*) NEAR/3 (sex* NEAR/1 (abuse* OR assault*))) OR (gender-based NEAR/3 (violen* OR abus*))) AND ab(maternal* or pregnan* or gestation* or prenatal* or perinatal* or antenatal* or postnatal* or postpartum* or "post-partum*" or "pre-partum*" or Peripartum* or puerperium* or puerperal* or Obstetric* or Parturition* or Lactat* or breastfeed* or breast feed* or milk expression* or ((bottle* or formula*) N/3 feed*) or Childbirth* or "child-bearing" or birthing or ((Child* or newborn* or infant*) N/3 birth*) or ((before or after) N/3 birth*) or "pre-birth*" or "post-birth*" or prepregnancy or "pre-pregnancy" or "post-pregnancy" or postpregnancy or preconception* or "pre-conception*" or postconception* or "post-conception*" or periconception* or "peri-conception*" or (expect* N/3 (mother* or mom? or child* or baby or babies)))
OR ((MAINSUBJECT.EXACT("Domestic violence") OR MAINSUBJECT.EXACT("Battered women")) AND (MAINSUBJECT.EXACT("Antenatal") OR MAINSUBJECT.EXACT.EXPLODE("Obstetrics") OR MAINSUBJECT.EXACT("Postpartum women") OR MAINSUBJECT.EXACT.EXPLODE("Pregnancy")))

Limit to 2000-current and to scholarly journals

Results: 1,109

**Appendix B: Data Collection Form**


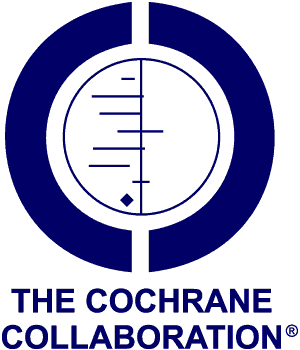
Data collection form

*Intervention review – Primary Studies*

Notes on using data extraction form:

1. Be consistent in the order and style you use to describe the information for each report.
2. Record any missing information as unclear or not described, to make it clear that the information was not found in the study report(s), not that you forgot to extract it.

| Date form completed *(dd/mm/yyyy)* |  |
| --- | --- |
| Initials of person extracting data |  |

1. Include any instructions and decision rules on the data collection form, or in an accompanying document. It is important to practice using the form and give training to any other authors using the form

**Preliminary Information**

| Study Title |  |
| --- | --- |
| Study ID *(surname of first author and year first full report of study was published e.g. Smith 2001)* |  |
| Reference citation |  |
|  |  |
|  |  |
| Publication type  *(e.g. full report, abstract, letter)* |  |
| Notes | |

**Study eligibility**

| Study Characteristics | Eligibility criteria  *(Insert inclusion criteria for each characteristic as defined in the Protocol)* | | Eligibility criteria met? | | | Location in text or source *(pg & ¶/fig/table/other)* |
| --- | --- | --- | --- | --- | --- | --- |
|  |  |  | Yes | No | Unclear |  |
| Type of study | Randomised Controlled Trial | |  |  |  |  |
|  | Other experimental design | |  |  |  |  |
|  | Prospective Cohort Study  Retrospective Cohort Study | |  |  |  |  |
|  | Other Observational Study Design | |  |  |  |  |
|  | Qualitative study – interpretive descriptive, case study, qualitative description, ethnography, narrative analysis | |  |  |  |  |
|  | Other qualitative design (specify): | |  |  |  |  |
|  | Other study design (specify) | |  |  |  |  |
| Participants | Note: primary eligible population includes women/individuals in the perinatal period (12 months preconception, pregnant, 12 months post-partum). | |  |  |  |  |
| Types of intervention | Note: Screening, or primary prevention interventions will be EXCLUDED | |  |  |  |  |
| Types of comparison |  | |  |  |  |  |
| Types of outcome measures | Note: Outcomes included: 1) Reducing IPV recurrence and associated harms and 2) Reducing adverse maternal and neonatal outcomes. | |  |  |  |  |
| INCLUDE | | EXCLUDE | | | | |
| Reason for exclusion |  | | | | | |
| Notes: | | | | | | |
|  |  |  |  |  |  |  |

**DO NOT PROCEED IF STUDY EXCLUDED FROM REVIEW**

Page Break

**Characteristics of included studies**

General Information

|  | **Descriptions as stated in report/paper** | **Location in text or source** *(pg & ¶/fig/table/other)* |
| --- | --- | --- |
| **Design** *(e.g. parallel, crossover, non-RCT)* |  |  |
| **Aim of study** *(e.g. efficacy, equivalence, pragmatic)* |  |  |
| **Author Names** |  |  |
| **Country of conduct** |  |  |
| **Publication Year** |  |  |
| **Sample Size Total** |  |  |
| **Start Date** |  |  |
| **End date** |  |  |
| **Duration of participation**  *(from recruitment to last follow-up)* |  |  |
| **Notes:** | | |

Participants

|  | Description  *Include comparative information for each intervention or comparison group if available* | Location in text or source *(pg & ¶/fig/table/other)* |
| --- | --- | --- |
| Antenatal, postpartum or perinatal period?  *The perinatal period is between 12 months before pregnancy to 12 months after delivery. The antenatal period is 12 months before delivery and postnatal period is 12 months after delivery.* |  |  |
| Population description (from which study participants are drawn) |  |  |
| Inclusion criteria for study’s participants |  |  |
| Exclusion criteria for study’s participants |  |  |
| Method of recruitment of participants  *i.e. phone, mail, clinic patients* |  |  |
| Baseline imbalances (if applicable) |  |  |
| Withdrawals and exclusions  *if not provided below by outcome* |  |  |
| Age  *Mean age of participants in years* |  |  |
| Gender  *Are only female, or both female and males included as participants?* |  |  |
| Race/Ethnicity  *Optional: Any noticeable race/ethnicity imbalance?* |  |  |
| Socioeconomic Characteristics  *If there is a main target socioeconomic class for the study, what is the primary socioeconomic status of the population?* |  |  |
| Subgroups reported |  |  |
| Notes: | | |

Intervention + Comparator Characteristics

*Copy and paste table for each intervention and comparison group*

**Intervention Group 1**

|  | Description as stated in report/paper | Location in text or source *(pg & ¶/fig/table/other)* |
| --- | --- | --- |
| Intervention Type  *Description of therapy or program ie. Cognitive behavioural therapy, education sessions, nursing home visitation, shelter and housing* |  |  |
| Why  *Describe any rationale, theory, or goal of the elements essential to the intervention.* |  |  |
| Number in intervention group  *specify whether no. people or clusters* |  |  |
| Target(s) for intervention *Which*  *Group(s) does the intervention target? i.e. Victim of IPV, family, couple, etc* |  |  |
| Setting of intervention (geographical)  *i.e Rural setting, urban setting* |  |  |
| Setting of intervention (clinical setting)  *Clinical or community setting? Specify exactly where (i.e. OB-hospital setting, At home, Online)* |  |  |
| Duration of intervention period  *How many days/months/years from recruitment to the last intervention session* |  |  |
| Timing  *frequency, duration of each episode Ie Once per month* |  |  |
| Length of Each Intervention Session  *How long each individual intervention session lasts i.e. 20-60 minutes per home visitation* |  |  |
| Delivery  *The medium of the intervention i.e. In person, online modules, 1-on-1 session, group sessions* |  |  |
| Provider  *Which member of the health team delivered the intervention? What profession?* |  |  |
| Provider Training    *What additional training did the provider receive to deliver the intervention? I.e. 10 hour IPV training module* |  |  |
| Co-interventions  *Were there additional interventions beyond the main intervention concurrently? i.e SSRIs, addiction therapy, social assistance, government programs.* |  |  |
| Economic information  Cost of intervention i.e. payment of healthcare provider. If specified, list numerical amount |  |  |
| Resource requirements  *Number of staff required, any equipment needed* |  |  |
| Personalization  *If intervention was planned to be tailored or adapted to each individual, describe how it was done, when and why.* |  |  |
| Modifications  *List any modifications to intervention that may have occurred during the study period. Explain why, when and how.* |  |  |
| Fidelity  *Strategies to maintain adherence or up-keep with intervention planned? Was intervention delivered as planned?* |  |  |
| Compliance *When applicable, extent in which participant following intervention program or treatment. Also known as "adherence". List as percentage* |  |  |
| Notes: | | |

Referral

|  | Description as stated in report/paper | Location in text or source *(pg & ¶/fig/table/other)* |
| --- | --- | --- |
| Healthcare provider referring    *Which member of the health team which referred the participant to the study i.e. OBGYN, Family doctor* |  |  |
| Method of referral/recruitment  *i.e. mail, phone, clinic visit (in-person), clinic visit (virtual)* |  |  |
| Referral Setting    *Clinical or community setting in which the referral took place i.e. OB-hospital setting, tertiary care* |  |  |
| Informed consent obtained? | Yes              No               Unclear |  |
| **IPV Identification** | | |
| If IPV was identified prior to referral, what was the tool for IPV identification?  *I.e. Hurt, Insult, Threaten, Scream (HITS) Woman Abuse Screen Tool (WAST)* |  |  |
| Was IPV identification done through **universal screening** or **case identification**? |  |  |
| Medium of IPV Identification  *i.e.tablet/computer, written, in-person questioning* |  |  |
| Healthcare Provider Identifying IPV    *Note, this can sometimes be different from the healthcare provider that provides the referral.* |  |  |
| Was IPV identification done via a self-reporting tool/method? | Yes              No |  |

Outcomes

*Copy and paste table for each outcome.*

**Outcome 1**

|  | Description as stated in report/paper | Location in text or source *(pg & ¶/fig/table/other)* |
| --- | --- | --- |
| Outcome category  *(1) Harms Associated with IPV or (2) Obstetrical Health Outcomes or both (1) and (2)* |  |  |
| Outcome Name |  |  |
| Is this outcome listed on the Outcome Definitions Table?  *If yes, proceed. If no, provide a brief definition and description of outcome* |  |  |
| Time points measured  *(specify whether from start or end of intervention)* |  |  |
| Effect Sizes  *Describe type of statistic reported and value i.e. confidence interval, p-value* |  |  |
| Conclusion  *Describe what key statements authors noted in conclusion statements about their intervention and its effectiveness* |  |  |
| Notes: | | |

Other

| **Study funding sources** *(including role of funders)* |  |  |
| --- | --- | --- |
| **Possible conflicts of interest** *(for study authors)* |  |  |
| **Ethical approval needed/ obtained for study** | Yes                  No           Unclear |  |
| **References to other relevant studies** |  |  |
| **Notes:** | | |

**Definitions**

| Assumed risk estimate | An estimate of the risk of an event or average score without the intervention, used in Cochrane 'Summary of findings tables'. If a study provides useful estimates of the risk or average score of different subgroups of the population, or an estimate based on a representative observational study, you may wish to collect this information. |
| --- | --- |
| Bias | A systematic error or deviation in results or inferences from the truth. In studies of the effects of health care, the main types of bias arise from systematic differences in the groups that are compared (selection bias), the care that is provided, exposure to other factors apart from the intervention of interest (performance bias), withdrawals or exclusions of people entered into a study (attrition bias) or how outcomes are assessed (detection bias). Reviews of studies may also be particularly affected by reporting bias, where a biased subset of all the relevant data is available. |
| Change from baseline | A measure for a continuous outcome calculated as the difference between the baseline score and the post-intervention score. |
| Clusters | A group of participants who have been allocated to the same intervention arm together, as in a cluster-randomised trial, e.g. a whole family, town, school or patients in a clinic may be allocated to the same intervention rather than separately allocating each individual to different arms. |
| Co-morbidities | The presence of one or more diseases or conditions other than those of primary interest. In a study looking at treatment for one disease or condition, some of the individuals may have other diseases or conditions that could affect their outcomes. |
| Compliance | Participant behaviour that abides by the recommendations of a doctor, other health care provider or study investigator (also called adherence or concordance). |
| Contemporaneous data collection | When data are collected at the same point(s) in time or covering the same time period for each intervention arm in a study (that is, historical data are not used as a comparison). |
| Controlled Before and After Study (CBA) | A non-randomised study design where a control population of similar characteristics and performance as the intervention group is identified. Data are collected before and after the intervention in both the control and intervention groups |
| Exclusions | Participants who were excluded from the study or the analysis by the investigators. |
| Imputation | Assuming a value for a measure where the true value is not available (e.g. assuming last observation carried forward for missing participants). |
| Integrity of delivery | The degree to which the specified procedures or components of an intervention are delivered as originally planned. |
| Interrupted Time Series (ITS) | A research design that collects observations at multiple time points before and after an intervention (interruption). The design attempts to detect whether the intervention has had an effect significantly greater than the underlying trend. |
| Post-intervention | The value of an outcome measured at some time point following the beginning of the intervention (may be during or after the intervention period). |
| Power | In clinical trials, power is the probability that a trial will obtain a statistically significant result when the true intervention effect is a specified size. For a given size of effect, studies with more participants have greater power. Note that power should not be considered in the risk of bias assessment. |
| Providers | The person or people responsible for delivering an intervention and related care, who may or may not require specific qualifications (e.g. doctors, physiotherapists) or training. |
| Quasi-randomised controlled trial | A study in which the method of allocating people to intervention arms was not random, but was intended to produce similar groups when used to allocate participants. Quasi-random methods include: allocation by the person's date of birth, by the day of the week or month of the year, by a person's medical record number, or just allocating every alternate person. |
| Reanalysis | Additional analysis of a study's results by a review author (e.g. to introduce adjustment for correlation that was not done by the study authors). |
| Report ID | A unique ID code given to a publication or other report of a study by the review author (e.g. first author's name and year of publication). If a study has more than one report (e.g. multiple publications or additional unpublished data) a separate Report ID can be allocated to each to help review authors keep track of the source of extracted data. |
| Sociodemographics | Social and demographic information about a study or its participants, including  economic and cultural information, location, age, gender, ethnicity, etc. |
| Study ID | A unique ID code given to an included or excluded study by the review author (e.g. first author's name and year of publication from the main report of the study). Although a study may have multiple reports or references, it should have one single Study ID to help review authors keep track of all the different sources of information for a study. |
| Theoretical basis | The use of a particular theory (such as theories of human behaviour change) to design the components and implementation of an intervention |
| Unit of allocation | The unit allocated to an intervention arm. In most studies individual participants will be allocated, but in others it may be individual body parts (e.g. different teeth or joints may be allocated separately) or clusters of multiple people. |
| Unit of analysis | The unit used to calculate N in an analysis, and for which the result is reported. This may be the number of individual people, or the number of body parts or clusters of people in the study. |
| Unit of measurement | The unit in which an outcome is measured, e.g. height may be measured in cm or inches; depression may be measured using points on a particular scale. |
| Validation | A process to test and establish that a particular measurement tool or scale is a good measure of that outcome. |
| Withdrawals | Participants who voluntarily withdrew from participation in a study before the completion of outcome measurement. |

Sources:

Cochrane Collaboration Glossary, 2010. Available from <http://www.cochrane.org/training/cochrane-handbook>.

Higgins JPT, Green S (editors). Cochrane Handbook for Systematic Reviews of Interventions Version 5.1.0 [updated March 2011]. The Cochrane Collaboration, 2011. Available from [www.cochrane-handbook.org](http://www.cochrane-handbook.org/).

Last JM (editor), A Dictionary of Epidemiology, 4^th^ Ed. New York: Oxford University Press, 2001.
